# Leveraging omic features with F3UTER enables identification of unannotated 3’UTRs for synaptic genes

**DOI:** 10.1038/s41467-022-30017-z

**Published:** 2022-04-27

**Authors:** Siddharth Sethi, David Zhang, Sebastian Guelfi, Zhongbo Chen, Sonia Garcia-Ruiz, Emmanuel O. Olagbaju, Mina Ryten, Harpreet Saini, Juan A. Botia

**Affiliations:** 1 Astex Pharmaceuticals, 436 Cambridge Science Park, Cambridge, UK; 2 Department of Neurodegenerative Disease, Institute of Neurology, University College London, London, UK; 3 Genetics and Genomic Medicine, Great Ormond Street Institute of Child Health, University College London, London, WC1E 6BT UK; 4 Verge Genomics, South San Francisco, CA 94080 USA; 5 NIHR Great Ormond Street Hospital Biomedical Research Centre, University College London, London, UK; 6 Department of Information and Communications Engineering, University of Murcia, Murcia, Spain

**Keywords:** Learning algorithms, Computational models, Data integration, Sequence annotation, Machine learning

## Abstract

There is growing evidence for the importance of 3’ untranslated region (3’UTR) dependent regulatory processes. However, our current human 3’UTR catalogue is incomplete. Here, we develop a machine learning-based framework, leveraging both genomic and tissue-specific transcriptomic features to predict previously unannotated 3’UTRs. We identify unannotated 3’UTRs associated with 1,563 genes across 39 human tissues, with the greatest abundance found in the brain. These unannotated 3’UTRs are significantly enriched for RNA binding protein (RBP) motifs and exhibit high human lineage-specificity. We find that brain-specific unannotated 3’UTRs are enriched for the binding motifs of important neuronal RBPs such as *TARDBP* and *RBFOX1*, and their associated genes are involved in synaptic function. Our data is shared through an online resource F3UTER (https://astx.shinyapps.io/F3UTER/). Overall, our data improves 3’UTR annotation and provides additional insights into the mRNA-RBP interactome in the human brain, with implications for our understanding of neurological and neurodevelopmental diseases.

## Abstract (web summary)

3’ untranslated regions (3’UTRs) play a crucial role in regulating gene expression, but our 3’UTR catalogue is incomplete. Here, the authors develop a machine learning-based framework to predict previously unannotated 3’UTRs in 39 human tissues.

## Introduction

The 3’UTRs of protein-coding messenger RNAs (mRNAs) play a crucial role in regulating gene expression at the post-transcriptional level. They do so by providing binding sites for *trans* factors such as RBPs and microRNAs, which affect mRNA fate by modulating subcellular localisation, stability and translation^1,2^. There is evidence to suggest that these RNA-based regulatory processes may be particularly important in large, polarised cells such as neurons. Recent studies have shown that transcripts which are highly expressed in neurons have both significantly longer 3’UTRs and higher 3’UTR diversity^3,4^. Furthermore, it has been shown that thousands of mRNA transcripts localise within subcellular compartments of neurons and undergo regulated local translation, allowing neurons to rapidly react to local extracellular stimuli^4–7^. Thus, there has been growing interest in the impact of 3’UTR usage on neuronal function in health and disease.

However, despite ongoing efforts to identify and characterise 3’UTRs in the human genome^8–11^, there is evidence to suggest that our current catalogue is incomplete^3,12–14^. Large-scale 3’end RNA-sequencing (RNA-seq) has identified a large number of novel polyadenylation (poly(A)) sites, many of which are located outside of annotated exons^12,13^. These insights are complemented by increasing recognition of the functional importance of transcriptional activity outside of known exons, particularly in human brain tissues^15–17^. This raises the possibility of developing new approaches for 3’UTR identification seeded from short-read RNA-seq data, but this is challenging, as evidenced by the shortcomings of transcript assembly tools to accurately annotate 3’UTRs^18–20^ and the subsequent development of alternative polyadenylation profiling tools^21^. The latter also has limitations, including the dependence on a priori gene annotations (e.g., TAPAS^22^, APAtrap^23^, DaPars^24^ and QAPA^25^) or assembled transcripts (e.g., ExUTR^26^ and Aptardi^27^) and the focus on identifying novel poly(A) sites within or in the vicinity of annotated 3’UTRs. This hinders their application to other transcriptionally active intergenic regions and so to the identification of unannotated 3’UTRs. Deep learning-based methods to predict poly(A) sites directly from DNA sequences e.g., APARENT^28^, DeepPASTA^29^, OmniPolyA^30^ and Leung et al.^31^, can alleviate this problem, but these tools do not incorporate transcriptomic data and only work on sequences of 206 nucleotides in length (with APARENT as an exception), which makes them unsuitable to identify tissue-specific and full-length unannotated 3’UTRs.

In this study, we present a machine learning-based framework, named F3UTER, which leverages both genomic and tissue-specific transcriptomic features to identify unannotated 3’UTRs. We apply F3UTER to RNA-seq data from Genotype-Tissue Expression Consortium (GTEx) to predict hundreds of unannotated 3’UTRs across a wide range of human tissues, with the highest prevalence discovered in the brain. We provide evidence to suggest that these unannotated 3’UTR sequences are functionally significant and have higher human lineage specificity than expected by chance. More specifically, we found brain-specific unannotated 3’UTRs were enriched for genes involved in synaptic function and interact with neuronal RBPs implicated in neurodegenerative and neuropsychiatric disorders. We release our data in an online platform, F3UTER (https://astx.shinyapps.io/F3UTER/), which can be queried to visualise unannotated 3’UTR predictions and the omic features used to predict them.

## Results

### Annotation-independent expression analysis suggests the existence of unannotated 3’UTRs in the human brain

**Fig. 1:**
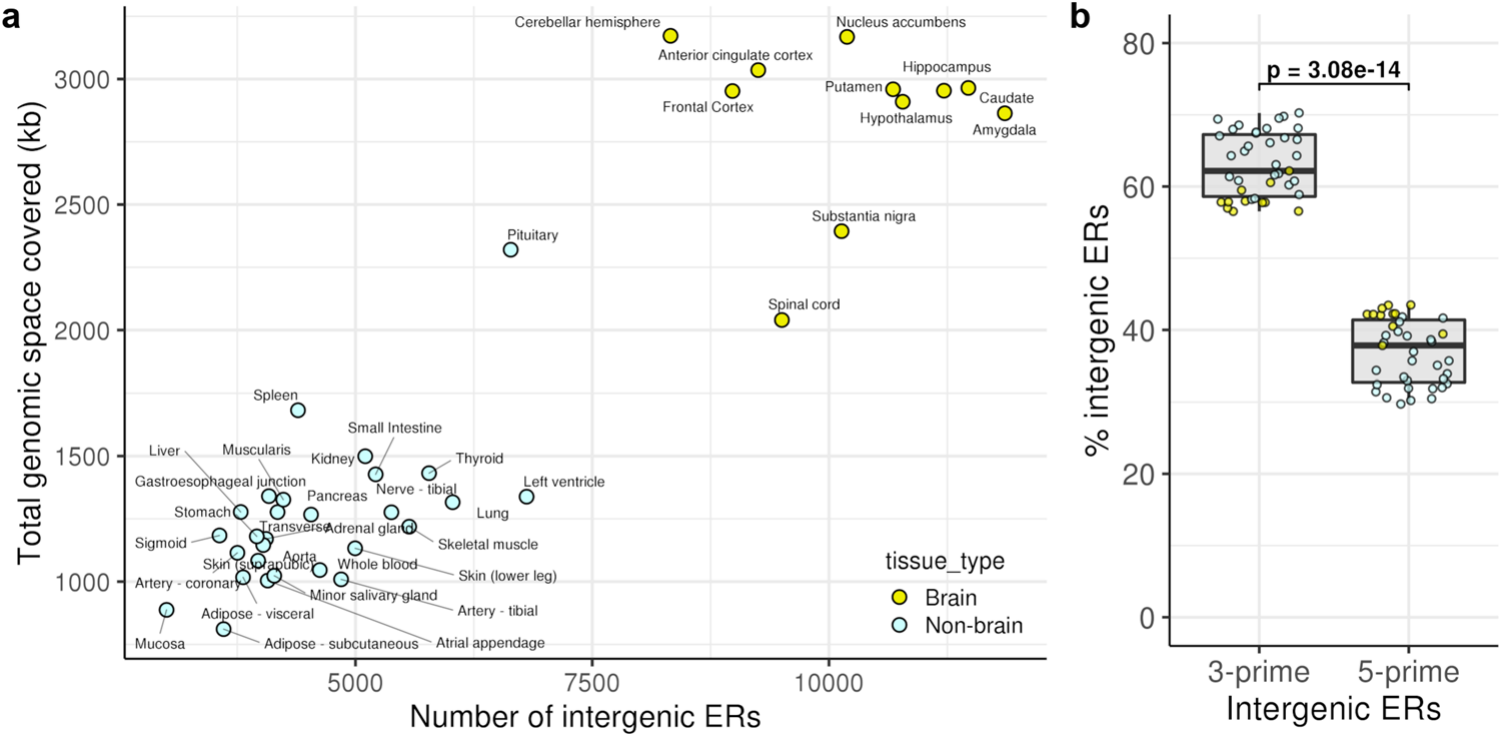
Enrichment of intergenic ERs across 39 GTEx tissues. **a** Scatter plot showing the number of intergenic ERs and their total genomic space covered in 39 human tissues. **b** Enrichment of intergenic ERs grouped by location with respect to their associated protein-coding gene. Box plots show the median value (middle line), 25th and 75th percentile (box), and 1.5 times the interquartile range (whiskers). Each data point in the box plot represents the proportion of total intergenic ERs in a tissue. *p:* *p* value calculated using two-sided Wilcoxon rank-sum test.

There is growing evidence to suggest that the annotation of the human brain transcriptome is incomplete and disproportionately so when compared to other human tissues^15–17^. We hypothesised that this difference may in part be attributed to an increased number of unannotated 3’UTRs in the human brain. To investigate this possibility, we analysed unannotated expressed regions of the genome (termed ERs) as previously reported by Zhang and colleagues^15^. These ERs were identified through annotation-independent expression analysis of RNA-seq data generated by GTEx with ER calling performed separately for 39 human tissues, including 11 non-redundant human brain regions. We focused on the subset of ERs most likely to be 3’UTRs, namely intergenic ERs which lie within 10 kb of a protein-coding gene ('Methods'). We found that these intergenic ERs were significantly higher in number ($p=1.19\times {10}^{-9}$, two-sided Wilcoxon rank-sum test) and total genomic space ($p=2.39\times {10}^{-9}$, two-sided Wilcoxon rank-sum test) in the brain compared to non-brain tissues (Fig. 1a). Furthermore, we discovered that intergenic ERs were significantly more likely to be located at 3’- rather than 5’-ends of their related protein-coding genes ($p=3.08\times {10}^{-14}$, two-sided Wilcoxon rank-sum test) (Fig. 1b), suggesting that a proportion of ERs detected in the human brain could represent unannotated 3’UTRs.

### Differentiating 3’UTRs from other expressed genomic elements is challenging

Given that existing studies indicate high levels of transcriptional noise and non-coding RNA expression in intergenic regions^32–35^, only some intergenic ERs are likely to be generated by unannotated 3’UTRs. This prompted us to develop a method to distinguish 3’UTRs from other transcribed genomic elements (non-3’UTRs) using short-read RNA-seq data. To achieve this aim, we first constructed a training set of known 3’UTRs (positive examples) and non-3’UTRs (negative examples) from Ensembl human genome annotation (v94). We obtained 17,719 3’UTRs and a total of 162,249 non-3’UTRs, consisting of five genomic classes: 21,798 5’UTRs, 130,768 internal coding exons (ICE), 3718 long non-coding RNAs (lncRNAs), 3819 non-coding RNAs (ncRNAs) and 2146 pseudogenes (Methods). For each of the positive and negative examples, we constructed a set of 41 informative omic features, which were broadly categorised as either genomic or transcriptomic in nature. Features calculated from genomic data included poly(A) signal (PAS) occurrence, DNA sequence conservation, mono-/di-nucleotide frequency, transposon occurrence and DNA structural properties. Features calculated from transcriptomic data included entropy efficiency of the mapped reads (EE, a measure of the uniformity of read coverage over a genomic region^36^), and percentage difference (PD, a measure of the absolute difference) between the reads mapped at the boundaries ('Methods'). To gain a better understanding of these features, we performed a univariate analysis to individually inspect the relationship between each feature and the genomic classes in our training dataset (i.e., 3’UTRs and all types of non-3’UTRs). Overall, while the genomic and transcriptomic features used had overlapping distributions amongst some genomic classes, each feature was significantly different when compared across all the genomic classes ($p < 2.2\times {10}^{-16}$, Kruskal–Wallis Test and two-sided proportion *Z*-test, Supplementary Fig. 1). This suggested that the features selected could be used to distinguish 3’UTRs from other genomic elements.

**Fig. 2:**
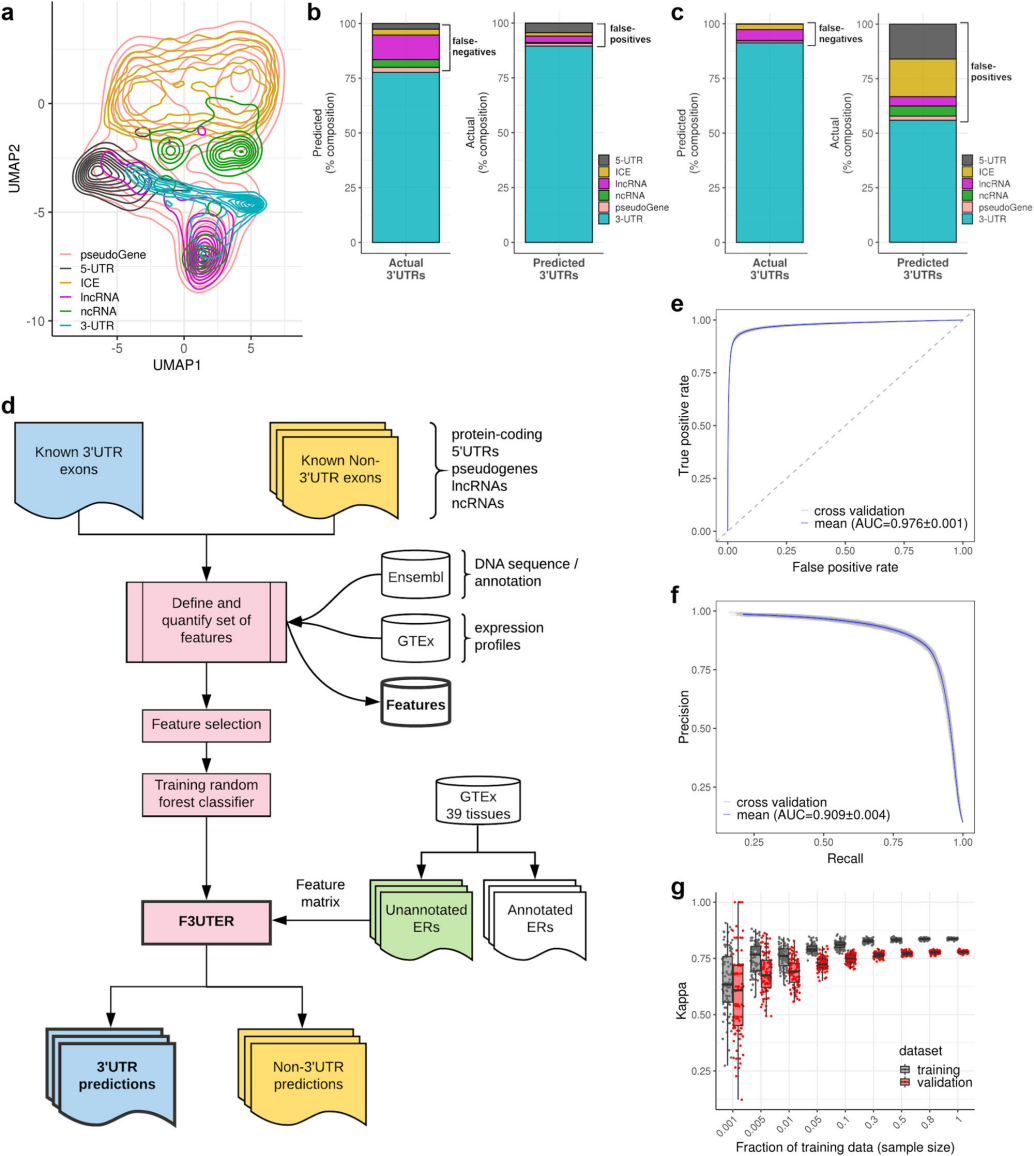
Classification of 3’UTRs from other transcribed elements in the genome. **a** UMAP representation of features, with elements labelled by genomic classes. **b** Classification of 3’UTRs using a multinomial random forest classifier. **c** Classification of 3’UTRs using an elastic net multinomial logistic regression model. **d** General framework of F3UTER: the core of the framework is a random forest classifier trained on omic features derived from known 3’UTRs and non-3’UTRs. The omic features are based on either genomic (DNA sequence) or transcriptomic (RNA-seq from GTEx) properties. To make predictions, genomic coordinates of ERs are given as input, from which a feature matrix is constructed. The output of the framework is ERs categorised into potential 3’UTRs and non-3’UTRs with their associated prediction probability scores. **e**, **f** ROC and precision-recall curves of F3UTER evaluated using fivefold cross-validation. **g** Bias-variance trade-off plot demonstrating the performance of F3UTER on training (*n* = 100) and validation (*n* = 100) datasets grouped by the sample size of the training data. Box plots show the median value (middle line), 25th and 75th percentile (box), and 1.5 times the interquartile range (whiskers).

To further investigate this for all 41 features across all six genomic classes, we applied a uniform manifold approximation and projection (UMAP)^37^ for dimensionality reduction into a 2D projection space. We found that while most 3’UTRs clustered separately from other classes within that space, some of them highly overlapped with other genomic classes such as lncRNAs, ICEs and 5’UTRs (Fig. 2a and Supplementary Fig. 2). These findings suggested that many unannotated 3’UTRs would be difficult to identify, and thus, may require an advanced classification approach based on machine learning to accurately distinguish them from other genomic elements.

### F3UTER accurately distinguishes 3’UTRs from other genomic elements across species

Next, we measured the predictive value of the omic features we had identified to distinguish between unannotated 3’UTRs and other expressed elements if used collectively. We trained a random forest multinomial classifier and evaluated its performance using fivefold cross-validation repeated 20 times ('Methods' and Supplementary Table 1). Consistent with the UMAP visualisation (Fig. 2a), we found that known 3’UTRs were most likely to be misclassified as lncRNAs (11%), followed by ncRNAs (4%), 5’UTRs (2.7%), ICEs (2.6%) and pseudogenes (2%) (Fig. 2b). On the other hand, false-positive 3’UTR predictions, which totalled 10%, were predominantly composed of known 5’UTRs (4%) and lncRNAs (3%). We compared this random forest multinomial classifier to an elastic net multinomial logistic regression model (Supplementary Table 2) and found that the random forest multinomial classifier had a significantly higher accuracy (76%; $p < 2.2\times {10}^{-16}$, two-sided Wilcoxon rank-sum test) and kappa (0.56; $p < 2.2\times {10}^{-16}$, two-sided Wilcoxon rank-sum test) in comparison to the multinomial logistic regression model (Supplementary Fig. 3). While the false-negative rate was higher (random forest classifier rate of 22%; logistic regression rate of 9%, Fig. 2c), importantly the random forest-based classifier reduced false-positive calling of 3’UTRs to 10% compared to 44% (17% ICEs, 16% 5’UTRs, 5% ncRNAs, 4% lncRNAs and 2% pseudogenes) using logistic regression. We also simplified the classification problem to a binary one and generated a second random forest classifier, aiming only to distinguish between 3’UTRs and non-3’UTRs. This resulted in the development of our final random forest classifier, **F**inding **3**’ Untranslated **E**xpressed **R**egions (F3UTER, Fig. 2d).

To assess F3UTER’s performance, we performed fivefold cross-validation (repeated 20 times) and calculated metrics such as accuracy, sensitivity, specificity, kappa, area under the ROC curve (AUROC) and area under the precision-recall curve (AUPRC). F3UTER achieved a mean accuracy of 0.96, sensitivity of 0.92, specificity of 0.96, kappa of 0.78, AUROC of 0.98 (Fig. 2e) and AUPRC of 0.91 (Fig. 2f) on the validation datasets (hold out) (Supplementary Table 3). We found that F3UTER performed similarly on both the training and validation datasets in the cross-validation (Fig. 2g). In addition, increasing the sample size of training data reduced the variability in model predictions and hence, made it more stable. Taken together, these findings suggested that we were not overfitting the classifier. Finally, we investigated the contributions of individual features towards the accuracy and node homogeneity (Gini coefficient, 'Methods') of 3’UTR classification. Interestingly, we found that features derived directly from sequence data (e.g., conservation and PAS) as well as from the transcriptomic data, namely mean-PD and mean-EE (Supplementary Fig. 4), most significantly contributed to the accuracy of F3UTER. This shows that F3UTER leverages both genomic and transcriptomic features to classify 3’UTRs, which would be expected to enable the identification of tissue-specific unannotated 3’UTRs.

**Fig. 3:**
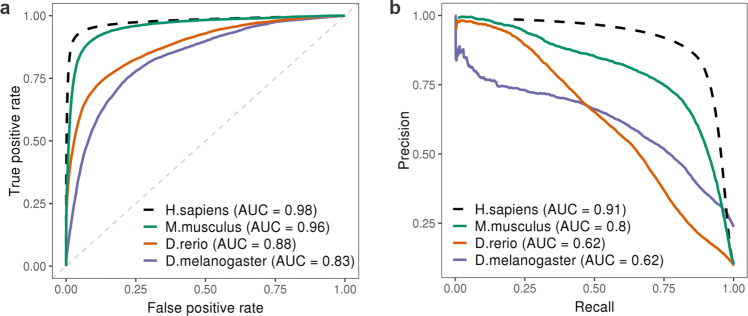
Performance of F3UTER in classifying 3’UTRs in non-human species. **a** ROC and (**b**) precision-recall curves of F3UTER evaluated by applying F3UTER to known 3’UTR and non-3’UTR regions from non-human species. Performance of F3UTER on human data evaluated using fivefold cross-validation is shown in black dotted lines for reference.

We next investigated whether F3UTER, a model trained on features derived from the human genome, can classify 3’UTRs in non-human species. For this analysis, we selected three commonly used model organisms: mouse (*Mus musculus*), fruit fly (*Drosophila melanogaster*) and zebrafish (*Danio rerio*). For each species, known 3’UTR and known non-3’UTR regions from Ensembl were used as the validation data for F3UTER (Methods and Supplementary Table 4), and omic features required by F3UTER were calculated. The genomic features were calculated from the DNA sequence of each species, whereas the transcriptomic features were calculated using publicly available RNA-seq data in the liver (for *M. musculus* and *D. rerio*) and midgut (for *D. melanogaster*) (Supplementary Table 5). We found that F3UTER performed remarkably well in classifying 3’UTRs in *M. musculus*, achieving an AUROC of 0.96 and AUPRC of 0.8 (Fig. 3 and Supplementary Table 6). Interestingly, given that F3UTER was trained on data that included expression features derived from human tissues, and PASs commonly found in mammals, F3UTER performed surprisingly well in *D. rerio* (AUROC = 0.88; AUPRC = 0.62) and *D. melanogaster* (AUROC = 0.83; AUPRC = 0.62), as these species are far apart from humans on the evolutionary tree. Overall, this analysis demonstrates that F3UTER can accurately classify 3’UTRs in *M. musculus*, and can potentially be used on data from other non-human species.

### Evaluation of F3UTER using 3’-end sequencing data validates unannotated 3’UTR predictions

We evaluated the performance of F3UTER using an independent dataset from Singh et al.^38^ consisting of both RNA-seq data and paired 3’-seq in four types of B cells (namely CD5 + B cells (CD5), germinal center B cells (GCB), memory B cells (MB) and naive B cells (NB); Supplementary Table 7). The latter, a form of 3’-end sequencing, was performed to identify poly(A) sites experimentally. Since poly(A) sites are present at the very end of 3’UTRs, unannotated 3’UTRs should overlap or be in the close vicinity of a poly(A) site. It should be noted that unlike the GTEx RNA-seq dataset which we used for our previous analyses and which consists of hundreds of samples for most tissues, the Singh et al. data consisted of a maximum of four RNA-seq samples. Since detecting unannotated ERs relies on averaging RNA-seq coverage across many samples to reduce the contribution of transcriptional noise to ER definition, calling ERs from ≤4 samples would likely result in inaccuracies at ER boundaries (Supplementary Fig. 5). Although this would be expected to significantly reduce the confidence in the detection of unannotated ERs and potentially underestimate the performance of F3UTER, the paired RNA-seq and 3’-seq nature of the Singh et al. data enabled us to confidently validate 3’UTR predictions using gold-standard experimental data.

**Fig. 4:**
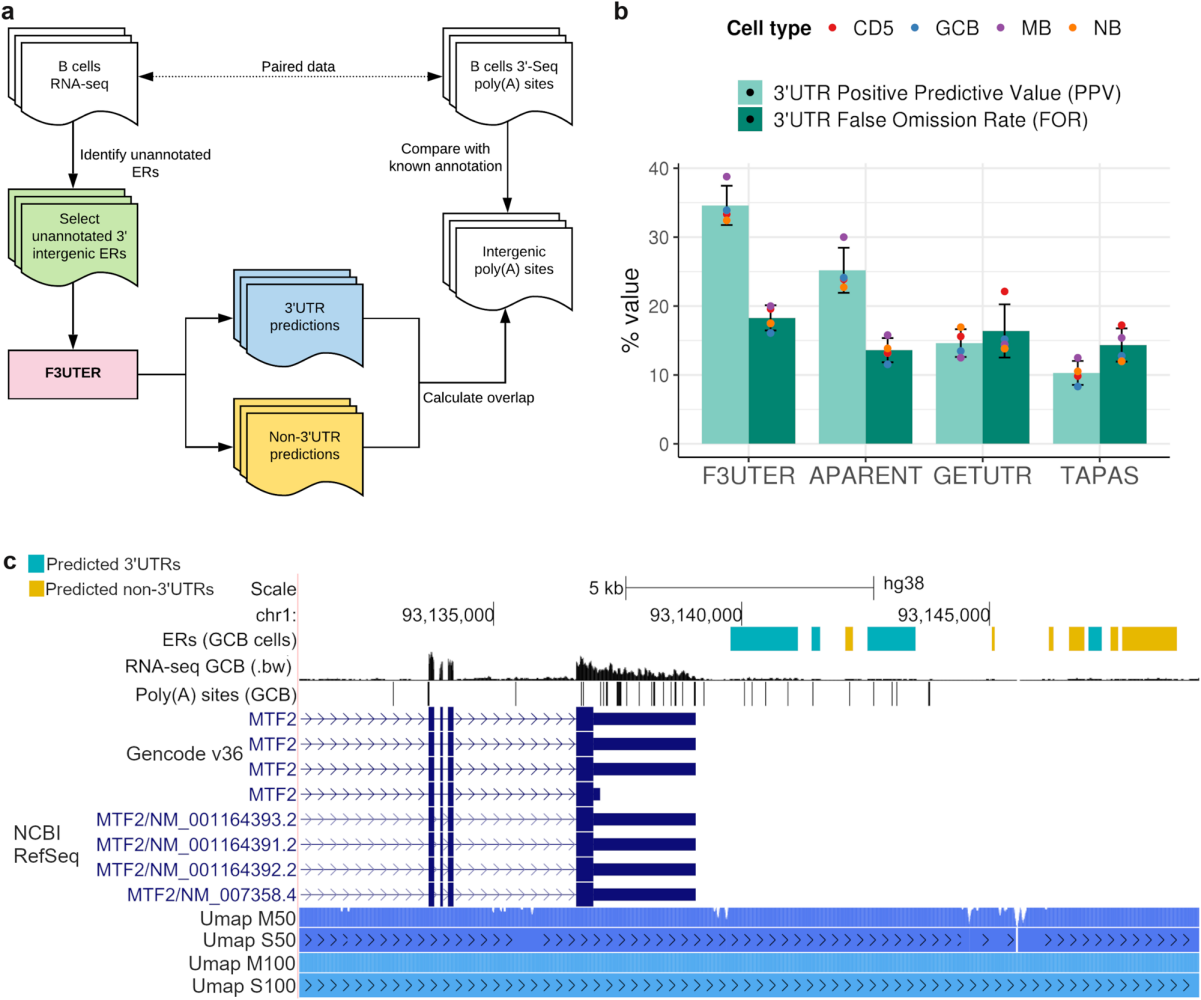
Evaluation of F3UTER predictions on an independent ER dataset using 3’-seq. **a** Schematic describing the framework of the process implemented to evaluate the performance of F3UTER on ERs in B cells. **b** Barplot showing the positive predictive value (PPV) and false omission rate (FOR) of 3’UTRs predicted by F3UTER, APARENT, GETUTR and TAPAS. The bars represent the average value across the four cell types (CD5, GCB, MB and NB), while the error bars show the standard deviation. The data points show the exact value of PPV and FOR in each cell type. **c** Genome browser view of the *MTF2* locus, showing unannotated 3’UTRs detected downstream of *MTF2* in GCB cells, RNA-seq expression in bigwig format (.bw), poly(A) sites in GCB, and the human genome mappability scores from UCSC (Umap). Umap S50 and S100: Single-read mappability for 50- and 100-mers; Umap M50 and M100: Multi-read mappability for 50- and 100-mers.

Using the RNA-seq data in B cells from Singh et al., we identified 3’ unannotated intergenic ERs following the pipeline used by Zhang et al.^15^. Then, we used F3UTER to predict unannotated 3’UTRs in these ER datasets, and compared these predictions to intergenic poly(A) clusters detected using paired 3’-seq (Fig. 4a). ERs predicted to be 3’UTRs which also overlapped with a poly(A) cluster were considered to be validated. Based on this approach, we calculated F3UTER’s positive predictive value (PPV, i.e., 3’UTR predictions overlapping a poly(A) cluster) and false omission rate (FOR, i.e., non-3’UTR predictions overlapping a poly(A) cluster) at varying prediction probabilities ('Methods' and Supplementary Fig. 6). As expected, we found that as we increased F3UTER’s prediction probability, its PPV increased, but the number of 3’UTR predictions decreased. Through visual inspection, we identified that a threshold of 0.6 for F3UTER’s prediction probability provided an optimal balance between PPV/FOR and the number of unannotated 3’UTR predictions. Therefore, we focused on only confident 3’UTR predictions, defined as those with a prediction probability of >0.6. As a reference, we noted that 88% of known 3’UTRs overlapped with a poly(A) cluster in B cells. We found that on average, 35% of 3’UTR predictions (with a prediction probability >0.6) were validated (Fig. 4b), as exemplified by the intergenic ER predicted to be an unannotated 3’UTR of the gene *MTF2* (Fig. 4c). The validation rate of 3’UTR predictions was 17.5-fold higher than that for randomly selected intergenic regions (2%, $p \, < \, 0.0001$, permutation test; Supplementary Fig. 7) and ~twofold higher than the validation rate of non-3’UTR predictions (18%, Fig. 4b). As would be expected, due to the inaccurate ER definitions in B cells as a result of small sample sizes, F3UTER’s performance in this setting was significantly lower as compared to that observed in cross-validation. However, we would expect F3UTER to perform better on ERs from GTEx as they were identified using hundreds of RNA-seq samples. Overall, these observations demonstrate the accuracy of F3UTER and show that it can effectively distinguish unannotated 3’UTRs from other functional genomic elements in the genome, even when applied to a small number of RNA-seq samples.

Lastly, we took advantage of paired RNA-seq and 3’-end datasets from Singh et al. to compare the performance of F3UTER with other tools to identify unannotated 3’UTRs from intergenic ERs. We compared F3UTER to a range of existing alternative polyadenylation profiling tools capable of predicting poly(A) sites, namely APARENT^28^, GETUTR^39^ and TAPAS^22^. We applied these tools to the same intergenic ER datasets generated from Singh et al. data to predict poly(A) sites within the ERs ('Methods'). If at least one of the predicted poly(A) site contained in an ER was within 30 nucleotides (nt) of a poly(A) cluster in the corresponding 3’-end data, it was considered a true positive. Whereas if an ER without any predicted poly(A) site overlapped a poly(A) cluster, it was considered a false negative. On average across the four cell types, APARENT, GETUTR and TAPAS achieved a PPV of 25%, 15% and 10%, and a FOR of 14%, 16% and 14%, respectively (Fig. 4b and Supplementary Table 8). However, we found that F3UTER not only achieved the highest PPV amongst all the tools (35%), but also gained the highest PPV-FOR ratio (1.9-fold). This analysis shows that F3UTER outperforms the three other tools in predicting unannotated 3’UTRs on these ER datasets.

### Applying F3UTER across 39 GTEx tissues identifies hundreds of unannotated 3’UTRs with evidence of the functional significance

**Fig. 5:**
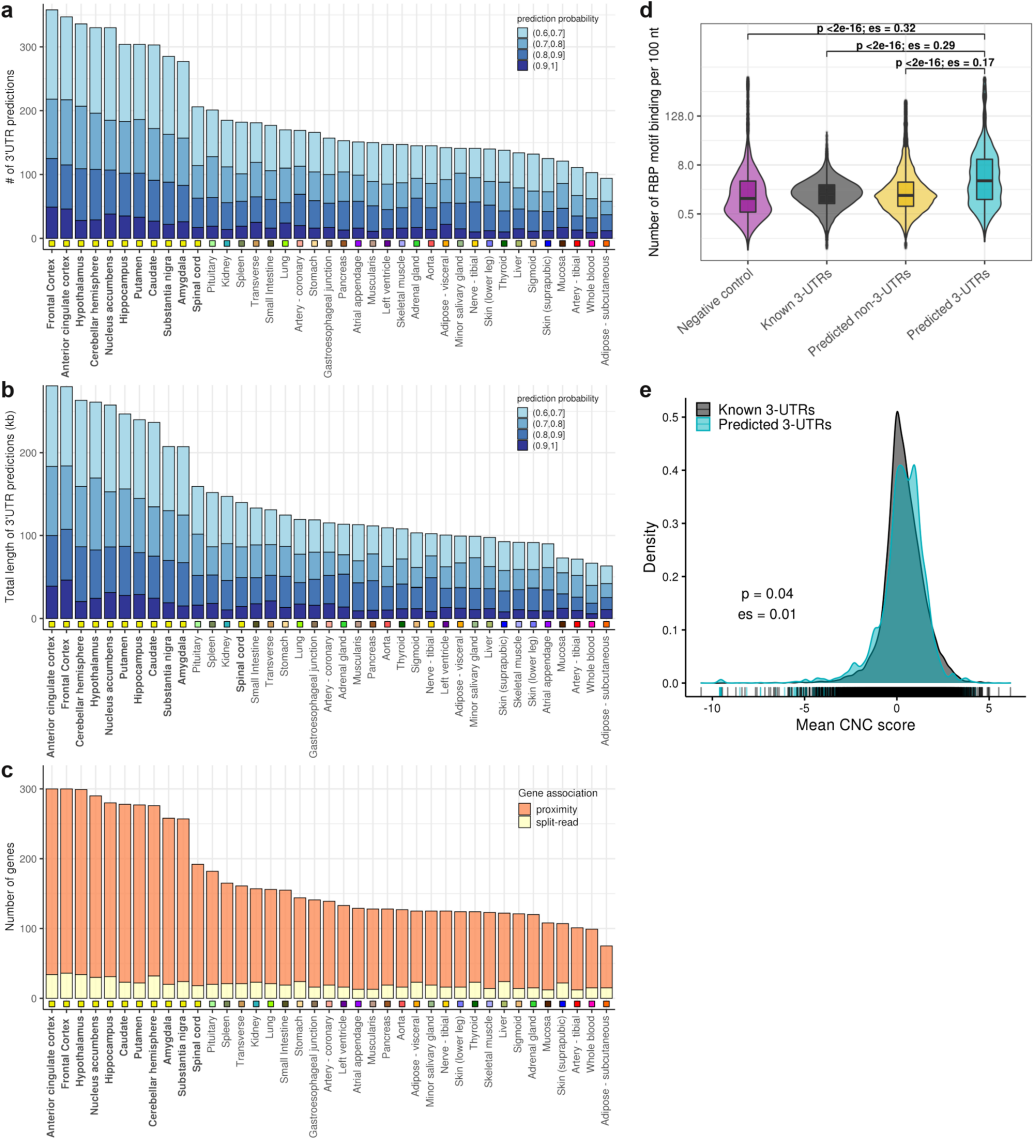
Unannotated 3’UTR predictions across 39 GTEx tissues. **a** Number of unannotated 3’UTRs predicted by F3UTER. **b** Total genomic space of unannotated 3’UTRs. The predictions are grouped and colour-coded based on their prediction probability score from F3UTER. **c** Number of genes associated with unannotated 3’UTRs, grouped by the type of gene association. In each barplot, tissues are sorted in descending order of the values plotted on the y-axis. The square boxes below the bars are colour-coded to group the tissues according to their physiology. **d** Violin plot comparing the RBP-binding density across known 3’UTRs (*n* = 14,924), predicted 3’UTRs (*n* = 7162), predicted non-3’UTRs (*n* = 57,688) and negative control (di-nucleotide shuffled sequences of predicted 3’UTRs, *n* = 6877). Only regions with RBP enrichment score greater than zero were displayed. Box plots show the median value (middle line), 25th and 75th percentile (box), and 1.5 times the interquartile range (whiskers). **e** Density distributions comparing the “constrained non-conserved” (CNC) scores between known (*n* = 15,279) and predicted 3’UTRs (*n* = 6522). *p:* *p* value of comparison calculated using two-sided Wilcoxon rank-sum test; es: Wilcoxon effect size (*r*).

We applied F3UTER to 3’ unannotated intergenic ERs identified by Zhang and colleagues^15^ in 39 tissues using RNA-seq data provided by GTEx. Similar to the ER datasets produced from Singh et al. data, we focused on confident 3’UTR predictions with a prediction probability of >0.6 (Supplementary Data 1). Across all tissues, we found that on average 7.8% of analysed ERs were predicted as unannotated 3’UTRs, with 8.3% being called in the brain (Supplementary Fig. 8). This equated to an average of 193 potentially unannotated 3’UTRs per tissue (ranging from 94 in adipose-subcutaneous to 358 in the frontal cortex, Fig. 5a), covering 63–280 kb of genomic space (mean across tissues =144 kb, Fig. 5b). By assigning predicted 3’UTRs to protein-coding genes either through the existence of junction reads or by proximity ('Methods' and Supplementary Fig. 9a), we estimated that 1563 distinct genes in total had unannotated 3’UTRs with an average of 171 genes per tissue (Fig. 5c). Of these 1563 genes across 39 tissues, 222 (14%) had at least one unannotated 3’UTR connected via junction reads, with a median distance of 2.7 kb between them (Supplementary Fig. 9b). The remaining 1341 genes (86%) were associated with unannotated 3’UTRs based on proximity, with a median distance of 2 kb between them. As expected, the number of predicted unannotated 3’UTRs was significantly higher in the brain relative to non-brain tissues (median values of 304 and 146 in the brain and non-brain tissues, respectively; $p=1.65\times {10}^{-6}$, two-sided Wilcoxon rank-sum test). This was associated with a significantly higher total genomic space (median values of 247 kb and 106 kb in the brain and non-brain tissues, respectively; $p=8.35\times {10}^{-9}$, two-sided Wilcoxon rank-sum test) and a higher number of implicated genes (median values of 278 and 126 in the brain and non-brain tissues respectively; $p=1.65\times {10}^{-6}$, two-sided Wilcoxon rank-sum test). These data suggest that incomplete annotation of 3’UTRs is present in all human tissues but is most prevalent in the brain.

Given that the intergenic ERs identified by Zhang et al. were defined based on Ensembl v92, we recognised that some of our predicted unannotated 3’UTRs could now be annotated in recent versions of reference gene annotations. Therefore, we assessed how many of our predicted unannotated 3’UTRs were annotated in Ensembl v104, Gencode v38 and RefSeq v109 (Curated). An unannotated 3’UTR was categorised as annotated if at least 50% of the unannotated 3’UTR overlapped with an annotated 3’UTR from the reference annotations. Of the 7528 predicted unannotated 3’UTRs across the 39 tissues, 1060 (14%; covering 0.92 Mb of genomic space and associated with 182 distinct genes) were annotated in at least one of the reference annotations (Supplementary Fig. 10a). We found that 90% of these 3’UTRs were associated with a protein-coding transcript and up to 77% were labelled as transcripts of the highest confidence (i.e., transcript support level = 1) in Ensembl v104 and Gencode v38 (Supplementary Fig. 10b, c), suggesting that these 3’UTRs are highly reliable annotations. Furthermore, 96% of the predicted unannotated 3’UTRs were associated with the same gene as annotated in Ensembl v104 and Gencode v38. We also compared our unannotated 3’UTRs to a previous study by Miura et al.^3^ where the authors identified unannotated 3’UTR extensions in 11 corresponding human tissues, including the brain. We found that 208 (3%) of our unannotated 3’UTRs (covering 188 kb of genomic space and associated with 99 distinct genes) were previously detected by Miura et al. in the corresponding tissues (Supplementary Fig. 10d). Overall, taking both reference gene annotations and Miura et al. data into account, 16% (1226) of our predicted 3’UTRs were annotated, covering 1.1 Mb of genomic space associated with 257 distinct genes (Supplementary Fig. 11). Thus, this analysis not only served to validate a significant proportion of our findings but indicated that many remain unknown.

Next, we investigated the functional significance of unannotated 3’UTRs by analysing their potential interaction with RBPs. This in silico analysis was performed because selective RBP binding at 3’UTRs is thought to be key in explaining the selection of alternate PASs and its impact on mRNA stability and localisation^40^. Using the catalogue of known RNA binding motifs from the ATtRACT database^41^, we examined the binding density of 84 RBPs across all unannotated 3’UTRs ('Methods'). Consistent with previous reports demonstrating higher RBP-binding densities in known 3’UTRs relative to other genomic regions^42^, we found that 3’UTR predictions were enriched for RBP-binding motifs compared to non-3’UTR predictions ($p \, < \, 2.2\times {10}^{-16}$, effect size (es) = 0.17, two-sided Wilcoxon rank-sum test) and negative control set ($p \, < \, 2.2\times {10}^{-16}$, es = 0.32, two-sided Wilcoxon rank-sum test) comprising of di-nucleotide shuffled sequences of 3’UTR predictions (Fig. 5d). Although non-3’UTR predictions exhibited some enrichment of RBP binding, we found that 34% of these regions had no RBP enrichment, which was ~fourfold higher than for the other sets (Supplementary Fig. 12a). Surprisingly, we noted that unannotated 3’UTRs were also enriched for RBP-binding motifs compared to known 3’UTRs ($p \, < \, 2.2\times {10}^{-16}$, es = 0.29, two-sided Wilcoxon rank-sum test, Fig. 5d), suggesting that these regions may be of particular functional significance. We repeated this analysis using a set of 97 RBPs from the CISBP-RNA database^43^ and found similar enrichment results (Supplementary Fig. 12b). To investigate this further, we leveraged constrained, non-conserved (CNC) scores^44^, a measure of human lineage specificity, to determine whether the unannotated 3’UTRs identified were of specific importance in humans. CNC score, a metric combining cross-species conservation and genetic constraint in humans, was used to identify and score genomic regions which are amongst the 12.5% most constrained within humans but yet are not conserved. We found that unannotated 3’UTRs exhibited significantly higher CNC scores compared to known 3’UTRs ($p=0.04$, es = 0.01, two-sided Wilcoxon rank-sum test, Fig. 5e). Thus, together our analyses suggested that unannotated 3’UTRs are not only functionally important but may be particularly crucial in human-specific biological processes.

### F3UTER identifies unannotated 3’UTRs of genes associated with synaptic function

**Fig. 6:**
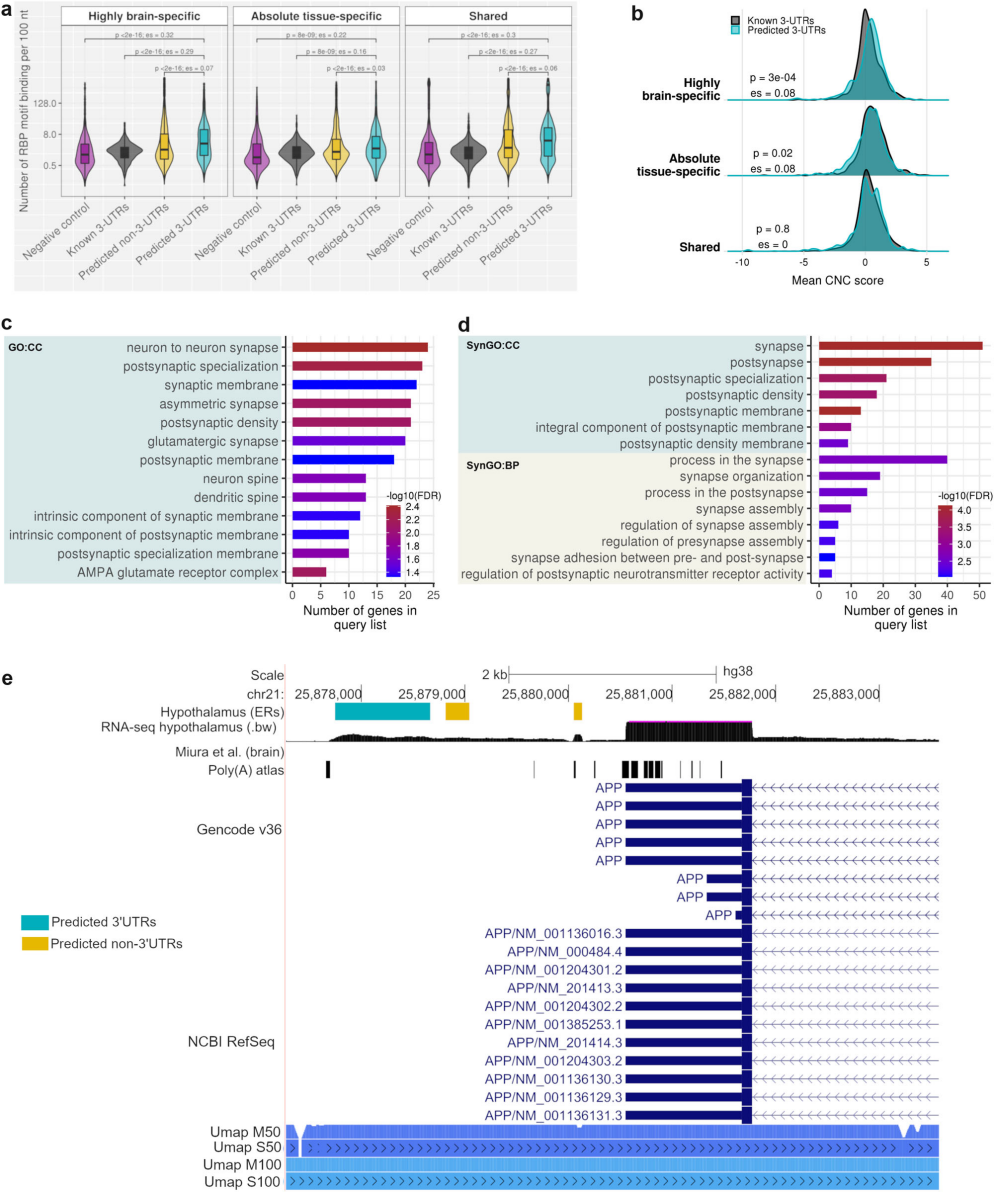
Functional significance of highly brain-specific unannotated 3’UTRs. **a** Violin plot comparing the RBP-binding density across known 3’UTRs, predicted 3’UTRs and predicted non-3’UTRs, categorised according to their tissue-specificity. Only regions with RBP enrichment score greater than zero were displayed. Box plots show the median value (middle line), 25th and 75th percentile (box), and 1.5 times the interquartile range (whiskers). See Supplementary Table 9 for sample size. **b** Density distributions comparing the 'constrained non-conserved' (CNC) scores between known 3’UTRs and predicted 3’UTRs, categorised according to their tissue-specificity. See Supplementary Table 10 for sample size. *p:* *p* value of comparison calculated using two-sided Wilcoxon rank-sum test; es: Wilcoxon effect size (*r*). **c** GO terms enriched amongst the list of genes associated with highly brain-specific unannotated 3’UTRs. CC cellular component. **d** SynGO terms over-represented in genes associated with highly brain-specific 3’UTRs. CC cellular component, BP biological process. **e** Genome browser view of the *APP* locus, showing unannotated 3’UTR detected downstream of *APP* in the hypothalamus, RNA-seq expression in bigwig format (.bw), poly(A) sites from the poly(A) atlas, and the human genome mappability scores from UCSC (Umap). Umap S50 and S100: Single-read mappability for 50- and 100-mers; Umap M50 and M100: Multi-read mappability for 50- and 100-mers.

Given the evidence for the functional importance of unannotated 3’UTRs predicted by F3UTER, we wanted to explore their biological relevance. To do this, we began by categorising all unannotated 3’UTRs into four sets based on their tissue-specificity: absolute tissue-specific (*n* = 308), highly brain-specific (*n* = 1571), shared (*n* = 2951) and ambiguous (*n* = 2698) ('Methods' and Supplementary Fig. 13). Using this non-redundant set of 3’UTRs, we found that on average, we extended the current annotation per gene by 710 nt in highly brain-specific (1.1× the known maximal 3’UTR length), 602 nt in tissue-specific (0.75× the known maximal 3’UTR length), and 713 nt in shared predictions (1× the known maximal 3’UTR length) respectively. Next, we repeated the RBP and CNC analysis for each category finding that all unannotated 3’UTR sets showed significant enrichment of RBP-binding motifs when compared not only to non-3’UTR predictions ($p \, < \, 2.2\times {10}^{-16}$, two-sided Wilcoxon rank-sum test), but also to known 3’UTRs ($p\le 8\times {10}^{-9}$, two-sided Wilcoxon rank-sum test), with the brain-specific set having the largest effect size (Fig. 6a and Supplementary Table 9). Focussing on CNC scores, we found that while shared unannotated 3’UTRs showed no significant difference in score compared to known 3’UTRs ($p=0.8$, two-sided Wilcoxon rank-sum test), absolute tissue-specific unannotated 3’UTRs trended to significance ($p=0.02$, two-sided Wilcoxon rank-sum test) and brain-specific unannotated 3’UTRs showed a prominent difference ($p=3\times {10}^{-4}$, es = 0.08, two-sided Wilcoxon rank-sum test) (Fig. 6b and Supplementary Table 10). Together, these observations lead us to conclude that highly brain-specific 3’UTR predictions were likely to be of most biological interest.

These observations raised the question of what types of genes are associated with highly brain-specific 3’UTR predictions. Interestingly, we found that genes linked to unannotated brain-specific 3’UTR predictions were significantly enriched for synapse-related GO terms (e.g., ‘neuron to neuron synapse’, ‘postsynaptic specialisation’ and ‘AMPA glutamate receptor complex’) (Fig. 6c and Supplementary Data 2). Moreover, genes linked to unannotated brain-specific 3’UTRs were more strongly associated with synapse compared to those linked with brain-specific non-3’UTR predictions (Supplementary Fig. 14). Using SynGO (the synaptic GO database^45^) to obtain more granular information, we found that genes associated with unannotated 3’UTRs were more significantly enriched for biological processes in the postsynapse ($q=3.2\times {10}^{-3}$) as compared to presynapse ($q=0.16$) (Fig. 6d and Supplementary Data 2). Furthermore, we found that 52 genes linked to unannotated brain-specific 3’UTRs were known to be associated with rare neurogenetic disorders ($p=0.28$, hypergeometric test), of which 10, more specifically, were associated with adult-onset neurodegenerative disorders ($p=0.09$, hypergeometric test) (Supplementary Table 11). For example, we detected an unannotated 3’UTR in the brain linked to the gene, *APP*, a membrane protein that when mutated gives rise to autosomal dominant Alzheimer’s disease and encodes for amyloid precursor protein, the main constituent of amyloid plaques^46^. We detected a 920-nt long brain-specific unannotated 3’UTR located 1.8 kb downstream of *APP* (Fig. 6e) and only 51 nt from an intergenic poly(A) site on the same strand as *APP* gene as reported by the poly(A) atlas. Other similar examples included the genes, *C19orf12*, *SCN2A, RTN2* and *OPA1* (Supplementary Figs. 15–18).

### Brain-specific unannotated 3’UTRs interact with RBPs implicated in neurological disorders

Next, we investigated the information content of brain-specific unannotated 3’UTRs by comparing RBP-binding enrichments between brain-specific and shared 3’UTR predictions (‘Methods’). By using shared 3’UTR predictions as the negative control, we removed RBPs associated with non-brain tissues and so identified RBP binding of greatest relevance to human brain function. This analysis identified eight RBPs with significantly enriched binding in the brain-specific unannotated 3’UTRs (${{{{{{\mathrm{adjusted}}}}}}\; p} \, < \, {10}^{-5}$) (Supplementary Table 12). We found that five of these RBPs were previously known to be associated with “mRNA 3’UTR binding” ($q=4.7\times {10}^{-9}$, Supplementary Data 3), including *TARDBP*, an RNA binding protein implicated in both frontotemporal dementia and amyotrophic lateral sclerosis^47^. Of the 80 gene targets that we identified for *TARDBP* through unannotated 3’UTRs, up to 41 had a *TARDBP*-binding site in their existing 3’UTR annotations, based on either computational scanning of *TARDBP* motif (44%, $p=1.7\times {10}^{-4}$, hypergeometric test) or *TARDBP* iCLIP experiments in brain (20%, $p=0.01$, hypergeometric test). However, this implied that 39 gene targets were not previously known to harbour *TARDBP*-binding motifs based on current annotation. Another RBP which was identified to be significantly enriched in brain-specific unannotated 3’UTRs was *RBFOX1*, a neuronal splicing factor implicated in the regulation of synaptic transmission^48^ and whose mRNA targets have been implicated in autism spectrum disorders^49^. We identified 87 gene targets with a predicted *RBFOX1*-binding motif within their associated unannotated 3’UTRs. Of these 87 genes, only 27 (31%, $p=0.01$, hypergeometric test) had a predicted *RBFOX1*-binding motif within their existing 3’UTRs, again implying that unannotated 3’UTRs provide valuable novel binding sites. Furthermore, SynGO enrichment analyses demonstrated that the target genes of *RBFOX1* were significantly enriched for processes in the synapse (e.g., 'synapse adhesion between pre- and post synapse', $q=3.1\times {10}^{-4}$; and 'regulation of presynapse assembly', $q=2\times {10}^{-3}$) (Supplementary Data 3), consistent with the previously known functions of *RBFOX1*^48^. These results show that the identification of brain-specific unannotated 3’UTRs can recognise additional genes within known regulatory networks, which can provide novel disease-relevant insights.

## Discussion

In this study, we generate a machine learning-based classifier, F3UTER, which leverages transcriptomic as well as genomic data to predict unannotated 3’UTRs. F3UTER outperforms elastic net logistic regression, whilst retaining its interpretability capabilities, and three other alternative polyadenylation profiling tools in identifying unannotated 3’UTRs. We apply F3UTER to transcriptomic data covering 39 human tissues studied within GTEx, enabling the identification of tissue-specific unannotated 3’UTRs. Using this large, public, short-read RNA-seq dataset, we predict unannotated 3’UTRs for 1563 genes, (equating to 5.6 Mb of genomic space in total across 39 tissues) and demonstrate that F3UTER can be successfully applied to human genomic regions from any tissue with existing bulk RNA-seq data. In fact, even though intergenic ERs in the four types of B cells were generated using ≤4 samples, we were able to validate 35% of the unannotated 3’UTR predictions using 3’-end sequencing data, showing that F3UTER can be a useful tool even for small RNA-seq datasets. Furthermore, it should be noted that F3UTER does not depend on ER datasets as input, but instead any set of interesting human genomic regions can be used. We note that F3UTER can also be applied to data from the mouse genome, which is evident from its high accuracy to classify known 3’UTRs in mice. Given the continued popularity and high availability of short-read RNA-seq data across tissues, cell types and disease states, we believe that (1) F3UTER could be applied more broadly to predict unannotated 3’UTRs and improve our understanding of 3’UTR diversity and usage, and (2) the set of omic features devised within this study could form the basis for other predictive models aimed at increasing the accuracy of human transcriptomic annotation.

We focus on F3UTER-predicted 3’UTRs in the human brain, which we find to be most prevalent when comparing predictions across all 39 human tissues. We believe that the higher frequency of incomplete 3’UTR annotation in human brain could be attributed to several factors including: (1) higher transcript diversity with many rare isoforms expressed in this tissue; (2) high cellular heterogeneity complicating detection of tissue-/cell-type-specific transcripts; (3) historically lower availability of human brain samples; and (4) reliance on post-mortem tissues, which suffer from RNA degradation resulting in decreased accuracy of transcript identification.

While we find that collectively the unannotated 3’UTRs predicted by F3UTER were significantly enriched for RBP binding and exhibited high human lineage specificity, the latter was primarily driven by brain-specific 3’UTR predictions. Overall, these findings suggest that predicted 3’UTRs are likely to be functionally important in the human genome. Moreover, these findings provide some explanation for the difficulties of identifying 3’UTRs through cross-species analyses particularly when considering brain-specific transcripts. Interestingly, we find that brain-specific unannotated 3’UTRs were enriched for binding of RBPs already implicated in neurological disorders, such as *TARDBP* and *RBFOX1*. Furthermore, genes linked to unannotated brain-specific 3’UTRs were significantly enriched for those involved in synaptic function, and although not a significant enrichment, some were already associated with rare neurogenetic and adult-onset neurodegenerative disorders.

Taken together, our results demonstrate that F3UTER not only improved 3’UTR annotation, but also identified unannotated 3’UTRs in the human brain which provided additional insights into the mRNA–RBP interactome with implications for our understanding of neurological and neurodevelopmental diseases. With this in mind, we note the growing interest in the role of 3’UTR-based mechanisms in translational regulation within complex, large, polarised cell types such as neurons^4,5,50,51^. Although increasing the use of single-nuclei RNA-seq, together with long-read RNA-seq will provide further insights into alternative 3’UTR usage and will impact the field considerably, these technologies still have significant limitations for the identification of rare transcripts. Therefore, we believe that F3UTER, which can effectively utilise existing short-read RNA-seq datasets, will be of interest to a wide range of researchers. Furthermore, we release our results through an online resource (F3UTER: https://astx.shinyapps.io/F3UTER/) which allows users to both easily query unannotated 3’UTRs and inspect the omic features driving the classifier’s prediction for an ER of interest.

## Methods

### ER data

We collected the set of intergenic ERs identified by Zhang and colleagues^15^ in 39 GTEx (v6) tissues, comprising of 11 non-redundant brain tissues and 28 non-brain tissues (total intergenic ERs = 9,339,770). Each ER was associated to the gene which connected to the ER via a junction read. In cases where no junction read was present, the nearest expressed gene (rpkm > 0.1) in the corresponding tissue was assigned to the ER. From this dataset, we selected intergenic ERs which were associated with protein-coding genes and were located within 10 kb of their associated genes, resulting in 240,529 ERs. Based on the location of intergenic ERs with respect to their associated genes, i.e., whether upstream or downstream, we annotated their orientation as 5’ (92,600 ERs) or 3’ (147,929 ERs) respectively. The total genomic space covered by these intergenic ERs was calculated by adding the length of all ERs in each tissue. To further remove ERs which were unlikely to be 3’UTRs, we selected 3’ intergenic ERs with a length ≤2 kb—which is the third quartile limit of known 3’UTR exon lengths. We also removed short ERs with length ≤40 nt for which feature calculation can be problematic. This resulted in a set of 94,922 intergenic 3’ ERs across all 39 tissues, and this set was used as input to F3UTER. Since the GTEx RNA-seq data used was un-stranded, the detected intergenic ERs had no strand information, and therefore, strand attribute was not used for orientation and filtering of intergenic ERs.

In this 3’ intergenic ER dataset, 5% of the ERs were connected to genes via junction reads, with a median distance of 2.5 kb between them. The remaining ERs were associated to genes based on proximity, i.e., the nearest expressed gene in the corresponding tissue, with a median distance of 2 kb between them. Overall, in the complete dataset (i.e., ER-gene associations based on both junction read and proximity), ERs were located at a median distance of 2 kb from their associated genes, with the majority of them (62%) located within 3 kb. We associated all ERs to a total of 5716 distinct genes across the 39 GTEx tissues, of which 834 genes (15%) were associated with at least one ER via junction reads. Amongst the ER-gene associations based on proximity, 93% of the nearest expressed genes were also the nearest gene. We found this percentage to be the same in the complete dataset. We identified that 446 genes associated with ERs via proximity (8% of total associated genes) overlapped another protein-coding gene on the same strand. Based on this we estimated that only 9% of the total ER-gene associations could be affected by overlapping genes.

### Assembling positive and negative 3’UTR learning datasets

For positive examples, we used known 3’UTRs, while for negative examples, we used regions in the genome which are known to be non-3’UTRs, namely 5’UTRs, internal coding exons (ICEs), lncRNAs, ncRNAs and pseudogenes. Ensembl human genome annotation (v94 GTF) was used to extract the genomic coordinates of these different genomic classes. For all classes in our training dataset, firstly, we selected high confidence annotations at the transcript level with transcript support level (TSL) = 1. Secondly, we collapsed and combined multiple transcripts associated with a single gene to make a consensus 'meta-transcript' per gene. This merged all the overlapping regions emerging from the same gene. Finally, we extracted exons with width >= 40 (nt) from these meta-transcripts to serve as learning examples.

To capture regions of 3’UTR exons, 5’UTR exons and ICEs, transcripts from protein-coding genes were selected. For ICE examples, transcripts with at least three coding exons were further selected (as transcripts with less than three exons would not contain an internal exon) and their first and last coding exons were removed to capture ICEs. To capture lncRNA, ncRNA and pseudogene exons, we selected annotations from the GTF file with the following gene biotypes:

- **lncRNA:** “non_coding”, “3prime_overlapping_ncRNA”, “antisense”, “lincRNA”, “sense_intronic”, “sense_overlapping”, “macro_lncRNA”
- **ncRNA:** “miRNA”, “misc_RNA”, “rRNA”, “snRNA”, “snoRNA”, “vaultRNA”
- **pseudogene:** “pseudogene”, “processed_pseudogene”, “unprocessed_pseudogene”, “transcribed_processed_pseudogene”, “transcribed_unitary_pseudogene”, “transcribed_unprocessed_pseudogene”, “translated_processed_pseudogene”, “unitary_pseudogene”, “unprocessed_pseudogene”, “TR_V_pseudogene”, “TR_J_pseudogene”, “rRNA_pseudogene”, “polymorphic_pseudogene”, “IG_V_pseudogene”, “IG_pseudogene”, “IG_J_pseudogene”, “IG_C_pseudogene”

### Calculating omic features

For each region in the training dataset, we calculated several genomic and transcriptomic-based features. Transcriptomic features were used to account for tissue-specific properties of transcribed elements in the genome.

### Genomic (sequence)-based features

- **Poly(A) signals (number of features,** ***n*** **=** 1): Previous studies have shown that 3’UTR sequences of most mammalian genes contain the consensus AAUAAA motif (or a close variant) 10–30 nt upstream of the poly(A) site^8^. These motif sites are recognised and bound by the cleavage and polyadenylation specificity factor (CPSF), and are referred to as polyadenylation signals (PASs). PASs are an important characteristic of 3’UTRs and are involved in the regulation of the polyadenylation process^8^. We used 12 commonly occurring PASs (AAUAAA, AUUAAA, AGUAAA, UAUAAA, AAUAUA, AAUACA, CAUAAA, GAUAAA, ACUAAA, AAUAGA, AAUGAA, AAGAAA)^9,12,52,53^ to construct a consensus position weight matrix (PWM). Each region was scanned for potential PWM matches and a binary outcome was reported i.e., whether the region contains a potential PAS or not. The “searchSeq” function (with min.score= “95%”) from the R package “TFBSTools” (v1.24.0)^54^ was used to detect PWM matches.
- **Mono- and di-nucleotide frequency (*n*** **=** **20):** The sequence composition in 3’UTRs, especially near the poly(A) sites has been shown to be important for polyadenylation^8,9,52^. The frequency probability of each mono-nucleotide (i.e. A, T G, C; *n* *=* 4) and di-nucleotide pair (*n* = 16; e.g. AA, AT, GC, GG) was calculated as the number of nucleotide occurrences divided by the length of the region.
- **DNA sequence conservation (*n*** = 1): Sequences of non-protein-coding transcripts and untranslated regions are poorly conserved compared to protein-coding sequences^55,56^. For every genomic position, we extracted the phastCons score of the human genome (hg38) across seven species pre-computed by the UCSC genome browser, and averaged it across the region to calculate mean sequence conservation score for each region.
- **Transposons (*n*** = 1): Previous studies have revealed that transposons are highly enriched within lncRNAs compared to protein-coding genes and other non-coding elements^57,58^. These transposable elements are considered to be the functional domains of lncRNAs. We calculated the total fraction of region covered with transposons—LINEs, SINEs, LTRs, DNA and RC transposons. The hg38 genomic coordinates of the transposable elements (Dfam v2.0) were downloaded from http://www.repeatmasker.org/species/hg.html.
- **DNA structural properties (*n*** = 16): The underlying sequence composition of a DNA molecule plays an important role in determining its structure. As a result, similar DNA sequences have a tendency to have similar DNA structures^59^. We calculated 16 properties of DNA structures which can be predicted from a nucleotide sequence based on previous experiments. To quantitatively measure a structural property from a nucleotide sequence, we used pre-compiled conversion tables downloaded from http://bioinformatics.psb.ugent.be/webtools/ep3/?conversion^60^. Depending on the structural property, we extracted scores for each di-nucleotide or tri-nucleotide occurrence in the sequence from the conversion tables, and averaged the scores across the region.

### Transcriptomic-based features

- **Entropy efficiency (*n*** = 1): We measured the uniformity of read coverage across a region using entropy efficiency, as described in Gruber et al.^36^. The entropy efficiency (EE) of a region (x) was calculated as, ${EE}(x)=-\frac{{\sum }_{i=1}^{n}p({x}_{i})\times {{\log }}(p({x}_{i}))}{{\log }(n)}$; $({x}_{i})=\frac{{x}_{i}}{\mathop{\sum }\limits_{j=1}^{n}{x}_{j}}$, where $n$ represents the length of the region and $p({x}_{i})$ is the read count at position $i$ divided by the total read count of the region. For each region, we calculated EE in 39 GTEx tissues and averaged it across all the tissues to obtain a baseline distribution of EE scores.
- **Percentage difference (*n*** = 1): We calculated the percentage difference (PD) between the read counts at the boundaries of a region. For read counts *r*_1_ and *r*_2_ measured at the boundaries of a region *x*, PD was calculated as: ${PD}\left(x\right)=\frac{|{r}_{1}-{r}_{2}|}{{{{{{{\mathrm{mean}}}}}}}\left({r}_{1},\,{r}_{2}\right)}$ × 100. For each region, we calculated PD in 39 GTEx tissues and averaged it across all the tissues to obtain a baseline distribution of PD scores.

### Univariate and multivariate analysis

For univariate analysis, we performed non-parametric Kruskal–Wallis test and two-sided proportion *Z*-test for continuous and categorical variables, respectively, to identify features with significant differences across all the genomic classes. We used UMAP^37^ to visualise all the features in two-dimensional space. The UMAP analysis was performed using the R package “umap” (v0.2.7.0) with default parameters. The clusters were visualised as a 2D density and a scatter plot. Each data point was labelled and coloured according to its genomic class.

To perform multivariate analysis, a feature matrix was generated where rows represented regions from the training dataset (*n* = 179,968), and columns represented the quantified features (*n* = 41). The features were scaled and centred in R using the preProcess function of R 'Caret' package (v6.0-85)^61^. The elastic net multinomial logistic regression model was trained using the 'glmnet' R package (v4.0-2)^62^ with the following parameters: family =  'multinomial', alpha = 0.5, nlambda = 25 and maxit = 10,000. The random forest multinomial classifier was trained within Caret using the “randomForest” package (v4.6-14)^63^ with default parameters (ntree = 500, nodesize = 1). We performed a fivefold cross-validation (repeated 20 times) to evaluate the performance of these multinomial classifiers, where the model was trained on 80% of the data (training dataset) and tested on 20% of the remaining data (validation dataset). Downsampling of the data was employed to correct for imbalance in the sample size of the classes. For each cross-validation run, we produced a confusion matrix for each prediction class using the Caret’s confusionMatrix function and computed the false-positive and negative rates. In addition, we report model’s overall Cohen’s kappa, which estimates the accuracy of a model compared to the expected accuracy and is a more accurate measure of performance for imbalanced datasets. These metrics were averaged across all the cross-validation runs for reporting purposes. With the elastic net logistic regression model, we found that known 3’UTRs were most likely to be misclassified as lncRNAs (4.98%), followed by ICEs (2.46%) and pseudogenes (0.88%). On the other hand, false-positive 3’UTR predictions, which totalled 44%, were predominantly composed of known ICEs (17.23%) and 5’UTRs (16.06%).

### F3UTER construction and evaluation

We designed F3UTER as a binary classifier to categorise an ER into a 3’UTR (positive) or a non-3’UTR (negative). This random forest classifier was implemented in R using Caret as the machine learning framework and 'randomForest' as the machine learning algorithm within Caret. The random forest classifier was trained using the default parameters (ntree = 500, nodesize = 1). We performed a fivefold cross-validation (repeated 20 times) to evaluate the performance of the F3UTER. For each cross-validation run, we calculated the performance metrics such as accuracy, kappa, sensitivity, specificity, ROC curve and precision-recall curve, using the caret’s confusionMatrix function. Variable importance was measured using mean decrease in accuracy and Gini coefficient, as natively reported by random forest. The Gini coefficient measures the contribution of variables towards homogeneity of nodes in the random forest tree. These metrics were averaged across all the cross-validation runs for reporting purposes. For bias-variance trade-off analysis, we trained F3UTER on sequentially increasing sample size of training data (0.1%, 0.5%, 1%, 5%, 10%, 30%, 50%, 80% and 100%), hence sequentially increasing the complexity of the model. For each sample size value, a fraction of the training data was randomly selected, and a fivefold cross-validation was performed which captured all the performance metrics for both the training and validation datasets. This process was repeated 20 times for each sample size. To make 3’UTR predictions on ER datasets, the classifier with the highest kappa statistic was selected from the cross-validation process.

### F3UTER evaluation in non-human species

For validation data in each species, we extracted known 3’UTR and known non-3’UTR (5’UTRs, ICEs, lncRNAs, ncRNAs and pseudogenes) regions from Ensembl (*Mus musculus* (GRCm38): v102; *Drosophila melanogaster* (BDGP6.32): v104; *Danio rerio* (GRCz10): v91). The processing of all genomic classes and the calculation of omic features for each region in the validation data was done in a similar way to F3UTER’s training data. Since F3UTER was trained on PASs found in mammals, each test region was scanned for the same set of PASs. PhastCons scores for *M. musculus* (mm10–60wayEuarchontoGlire) and *D. melanogaster* (dm6–27way) were downloaded from the UCSC genome browser. The genomic coordinates of transposable elements for all three non-human species were downloaded from http://www.repeatmasker.org/species/hg.html. Since pre-computed phastCons scores were not available for *D. rerio*, and pre-computed tables of DNA structural properties were not available for all three non-human species, we constructed two additional F3UTER models for this analysis: (1) trained without DNA structural properties and sequence conservation—applied to validation data from *D. rerio*; and (2) trained without DNA structural properties—applied to validation data from *M. musculus* and *D. melanogaster*.

The transcriptomic features were calculated using publicly available RNA-seq data in liver and midgut (details reported in Supplementary Table 5). The RNA-seq reads were downloaded from SRA using SRA-Toolkit (v2.10.0) in fastq format and were aligned to the respective genomes using STAR^64^ (v2.5). The reads were allowed to map to a maximum of one locus (--outFilterMultimapNmax 1). The resulting aligned reads were then converted into bigwig signals for calculating coverage over test regions. We also used kallisto^65^ (v0.45.0) to quantify gene abundance in transcripts per million (TPM). Known 3’UTR and non-3’UTR regions associated with genes not expressed (TPM$\le 0.1$) in the corresponding tissues (i.e., liver for *M. musculus* and *D. rerio*, and midgut for *D. melanogaster*) were removed from the validation data. This resulted in a total of 190,310, 108,786 and 47,517 test regions in *D. rerio*, *M. musculus* and *D. melanogaster*, respectively (Supplementary Table 4). The performance of F3UTER on these test regions was evaluated using performance metrics, such as accuracy, sensitivity, specificity, ROC curve and precision-recall curve.

### Validation of 3’UTR predictions using 3’-end sequencing data

Previously published RNA-seq and its corresponding 3’-end sequencing data from Singh et al.^38^ in four types of B cells (CD5 + B cells (CD5), germinal center B cells (GCB), memory B cells (MB) and naive B cells (NB)) was used for validating 3’UTR predictions (Supplementary Table 7). The RNA-seq reads were downloaded from SRA using SRA-Toolkit (v2.10.0) in fastq format and were aligned to the human genome (hg38) using STAR v2.5. The reads were allowed to map to a maximum of one locus (--outFilterMultimapNmax 1). The resulting aligned reads were then converted into bigwig signals and 3’ intergenic ERs were identified using the pipeline detailed in Zhang et al.^15^—which is compiled into a R package (ODER: https://github.com/eolagbaju/ODER). Multiple replicates were used for ER calling. Candidate 3’ intergenic ERs were processed and selected in the same way as ERs from GTEx. This resulted in 726 ERs in CD5 cells, 1158 ERs in GCB cells, 624 ERs in MB cells and 985 ERs in NB cells. Omic features required by F3UTER were then calculated for these 3’ intergenic ERs.

Processed poly(A) site clusters for 3’-end data associated with the RNA-seq samples from Singh et al. were downloaded from the poly(A) atlas^13^. Poly(A) site clusters from multiple replicates were unified and collapsed to produce a non-overlapping set. These poly(A) site clusters were compared to Ensembl human genome annotation (v92) to identify sites which occur within the intergenic regions. F3UTER was applied to 3’ intergenic ERs produced from Singh et al. data and the resulting predictions were compared to intergenic poly(A) site clusters to calculate their overlap. Predictions with at least a 1 base overlap with a poly(A) site were considered to be overlapping. Positive predictive value (PPV) was calculated as the number of 3’UTR predictions overlapping a poly(A) site divided by the total number of 3’UTR predictions ($\frac{{{{{{{\mathrm{true}}}}}}\; {{{{{\mathrm{positives}}}}}}}}{{{{{{{\mathrm{true}}}}}}\; {{{{{\mathrm{positives}}}}}}}+{{{{{{\mathrm{false}}}}}}\; {{{{{\mathrm{psoitives}}}}}}}}$). Likewise, false omission rate (FOR) was calculated as the number of non-3’UTR predictions overlapping a poly(A) site divided by the total number of non-3’UTR predictions ($\frac{{{{{{{\mathrm{false}}}}}}\; {{{{{\mathrm{negatives}}}}}}}}{{{{{{{\mathrm{false}}}}}}\; {{{{{\mathrm{negatives}}}}}}}+{{{{{{\mathrm{true}}}}}}\; {{{{{\mathrm{negatives}}}}}}}}$). A two-sided permutation test was performed to inspect if the observed overlap between 3’UTR predictions and intergenic poly(A) sites is more than what we would expect by random chance. Using BEDTOOLS^66^ (v2.29.2), the locations of 3’UTR predictions were shuffled in the intergenic genomic space on the same chromosome, hence generating random intergenic ERs with length, size and chromosome distribution similar to 3’UTR predictions in B cells. To shuffle the locations within the intergenic space, we excluded the genomic space covered by genes (all Ensembl biotypes) and intergenic ERs (both 3’ and 5’) in the respective B-cells data. The overlap between these randomly generated intergenic ERs and poly(A) sites was then calculated, and this process was repeated 10,000 times to produce a distribution of expected overlap. The *p* value was calculated as $\frac{x}{N}$, where $x$ is the number of times the expected overlap was greater than the observed overlap, and $N$ is the total number of permutations. The *z*-score was calculated as $\frac{{O}_{{obs}}-{O}_{{perm}}}{{{SD}}_{{perm}}}$, where ${O}_{{obs}}$ represents the observed overlap, ${O}_{{perm}}$ is the median of the permuted overlap, and ${{SD}}_{{perm}}$ is the standard deviation of the permuted distribution.

### Comparison to alternative polyadenylation profiling tools

F3UTER was compared to other alternative polyadenylation profiling tools using two metrics: PPV and FOR. TAPAS^22^ was included in this benchmarking study because it was identified as the top-performing method for predicting poly(A) sites in a previous benchmarking study^21^ comparing all alternative polyadenylation profiling tools using RNA-seq data. We also included APARENT^28^ (a recently published convolutional neural network) in our benchmarking analysis. This is because we noted that Chen and colleagues^21^ did not consider 3’UTR identification tools based on deep learning applied to DNA sequences.

### APARENT analysis

APARENT (APA REgression NeT)^28^ is a deep neural network trained to predict alternative polyadenylation in human 3’UTRs. We used APARENT’s poly(A) detection model (aparent_large_lessdropout_all_libs_no_sampleweights.h5) downloaded from APARENT’s GitHub repository (https://github.com/johli/aparent), which can predict the locations of poly(A) sites from DNA sequence. APARENT was applied on the DNA sequence of ER datasets from Singh et al. using default parameters. For each ER sequence, APARENT either predicted one or more poly(A) site(s), or no poly(A) site was predicted. ER sequences in which no poly(A) site was predicted by APARENT were considered to be non-3’UTR predictions.

### GETUTR analysis

GETUTR (Global estimation of the 3′ UTR landscape based on RNA-seq)^39^ predicts poly(A) cleavage sites in 3’UTRs using RNA-seq data. GETUTR’s source code (v2.0.0) was downloaded from http://big.hanyang.ac.kr/GETUTR/download.htm. GETUTR requires a refFlat reference file from which the genomic coordinates of 3’UTRs are extracted, and RNA-seq mapped reads in BAM format. We provided ERs to GETUTR as 3’UTRs in refFlat files and RNA-seq mapped reads from multiple replicates were pooled together. GETUTR was run with default parameters. For each ER, GETUTR either predicted one or more poly(A) site(s), or no poly(A) site was predicted. It should be noted that in many cases, GETUTR also predicted poly(A) sites outside the genomic coordinates of the input ERs. However, we followed a lenient approach and if such predicted poly(A) site overlapped with a true poly(A) site from 3’-end data, it was considered a true positive. ERs in which no poly(A) site was predicted by GETUTR were considered to be non-3’UTR predictions.

### TAPAS analysis

TAPAS (Tool for Alternative Polyadenylation Site analysis)^22^ is a programme to detect novel alternative polyadenylation sites from RNA-seq data. TAPAS’s model to find novel alternative polyadenylation sites (APA_sites_detection) was downloaded from its GitHub repository: https://github.com/arefeen/TAPAS. Similar to GETUTR, TAPAS requires a reference annotation file in refFlat format, and a coverage file for RNA-seq mapped reads. We provided ERs to TAPAS as 3’UTRs in refFlat files and coverage was calculated using pooled RNA-seq mapped reads. TAPAS was run with default parameters. For each ER, TAPAS either predicted one or more novel poly(A) site(s), or the original end of the ER was output. ERs for which the original end was output were considered to be non-3’UTR predictions.

### 3’UTR predictions in 39 GTEx tissues

A feature matrix of 3’ intergenic ERs was generated in each tissue. F3UTER was applied to each matrix to categorise intergenic ERs into 3’UTR (prediction probability $$ > 0.60$$) and non-3’UTR (prediction probability $$\le\! 0.60$$) predictions. Out of 94,922 3’ intergenic ERs across 39 tissues which were input to F3UTER, 7528 were predicted as 3’UTRs and 87,394 as non-3’UTRs. Of the predicted 3’UTRs, 811 (11%) were connected to genes via junction reads, with a median distance of 2.7 kb between them. Whereas the remaining predicted 3’UTRs were associated to genes based on proximity, with a median distance of 2 kb between them. Overall, a majority of the predicted 3’UTRs (64%) were located within 3 kb of their associated genes. Predicted 3’UTRs across all tissues were associated with 1563 distinct genes, of which 222 genes (14%) were connected to at least one predicted 3’UTR via junction reads. Of these 1563 genes, we identified that 120 genes associated with ERs via proximity (8%) overlapped another protein-coding gene on the same strand. Based on this we estimated that 7.5% of the total predicted 3’UTR-gene associations could be affected by overlapping genes. For each tissue, the lengths of the 3’UTR predictions were added to calculate their total genomic space (in kb). To compare brain and non-brain tissues, a two-sided Wilcoxon rank-sum test was applied to statistically compare the associated numbers between the two groups. The 3’UTR predictions were compared to gene annotations from Ensembl v104, Gencode v38 and RefSeq Curated v109. The comprehensive gene annotation file for reference chromosomes was used for Genocde, while the 3’UTRs for RefSeq were retrieved from the UCSC Table Browser. A 3’UTR prediction was considered detected if at least 50% of its region overlapped with an annotated 3’UTR from the reference gene annotations. The genomic coordinates of unannotated 3’UTR extensions identified by Miura et al.^3^ were extracted from the supplementary files provided with their study. These coordinates were mapped to hg19 and therefore were converted to hg38 for comparison. We considered all categories of predictions annotated by Miura et al. (candidate, confident and precise regions) in 11 human tissues (adipose, adrenal, brain, colon, heart, kidney, liver, lung, skeletal muscle, thyroid and white blood cells) corresponding to our set of GTEx tissues.

To explore the biological relevance of 3’UTR predictions, they were categorised into four groups based on their tissue-specificity: absolute tissue-specific, highly brain-specific, shared and ambiguous. To do such categorisation, the genomic coordinates of ER predictions were compared across the 39 tissues. An ER which did not overlap any other ER across the tissues was labelled as “absolute tissue-specific” or present in only one tissue (*n* = 7337). On the other hand, for an ER which overlapped (≥1 nt) ERs from other tissues, we calculated the proportion of brain tissues in which the ER was detected. If more than 75% of the tissues were brain-related, the ER was labelled as “highly brain-specific” (*n* = 21,111). From the remaining data, ERs detected in at least five tissues, with their start and end coordinates within a 10-nt window, were labelled as “shared” (*n* = 31,516). All the remaining ERs which did not fall in any of the above categories were labelled as “ambiguous” (*n* = 34,958).

### RBP and CNCR analysis

For the set of known 3’UTRs, we selected annotated 3’UTRs from Ensembl v94 which were “expressed” (mean coverage ≥5) in at least one of the 39 GTEx tissues. To do this, we calculated mean coverage of each annotated 3’UTR region using the GTEx RNA-seq data (v6). Annotated 3’UTRs with mean coverage of less than 5 reads in all 39 tissues were removed, resulting in 16,407 known 3’UTRs. We further categorised this known set of 3’UTRs into three groups: absolute tissue-specific, highly brain-specific and shared. 3’UTRs which were expressed in only one tissue were labelled as 'absolute tissue-specific' (*n* = 739). For 3’UTRs expressed in multiple tissues, we calculated the proportion of brain tissues in which the 3’UTR was expressed. If more than 75% of the tissues were brain-related, the 3’UTR was labelled as 'highly brain-specific' (*n* = 733). From the remaining data, 3’UTRs which were expressed in at least five tissues were labelled as 'shared' (*n* = 13,652). To scan genomic regions for RBP binding, we extracted the DNA sequence (hg38) of regions in predicted 3’UTRs, predicted non-3’UTRs and known 3’UTRs. The set of negative control sequences was obtained by shuffling the predicted 3’UTR sequences while preserving the di-nucleotide frequency, using the universalmotif R package (v1.4.10; method = “euler”).

The position weight matrices (PWMs) of RBP-binding motifs in humans were collected from the ATtRACT database^41^. Motifs with less than 7 nt in length and with a confidence score of less than one, were removed to reduce false positives in the motif matches. To remove redundancy between multiple motifs of a RBP, we further selected the longest available motif. This resulted in 84 unique PWMs, which were then used for identifying potential RBP-binding using tools from the MEME suite (v5.1.1)^67^. Curated RBP motifs from the CISBP-RNA database (*n* = 97) were retrieved from the MEME motif database (Homo_sapiens.dna_encoded_cisbp_rna). We used FIMO^68^ with a uniform background to scan query sequences for potential RBP motif matches. For each RBP motif and query sequence pair, we calculated normalised counts as the number of motif matches (with $p \, < \, {10}^{-5}$) per 100 nt of query sequence. To summarise this analysis, we then calculated an overall RBP-binding score for each query sequence by adding the normalised counts across all the RBPs. We used AME^69^ with default parameters to compare binding enrichment of RBPs between highly brain-specific (query) and shared 3’UTR predictions (control). RBP motifs with an enrichment ${{{{{{\mathrm{adjuested}}}}}}\; p}-{{{{{{\mathrm{value}}}}}}} \, < {10}^{-5}$ were considered to be significantly over-represented in highly brain-specific 3’UTR predictions compared to shared 3’UTR predictions. We used RBP motif scanning results from FIMO to identify unannotated 3’UTRs with a predicted *TARDBP* and *RBFOX1* binding. The existing 3’UTRs of genes associated with these unannotated 3’UTRs were scanned for *TARDBP* and *RBFOX1* motifs in a similar way using FIMO. Binding sites of *TARDBP* identified using iCLIP technology were extracted from the POSTAR2 database^70^. We downloaded all the curated RBP-binding sites in the human genome from POSTAR2, and selected *TARDBP* sites in the brain, which resulted in 133,037 binding sites from multiple iCLIP experiments. These binding sites were then collapsed and unified, resulting in a set of 118,157 non-redundant TARDBP-binding sites.

The CNC scores, as reported by Chen et al.^44^, were used to quantify the occurrence of CNCRs within unannotated 3’UTRs. We extracted the CNC score for each 10-nt window and averaged it across the query region to calculate a mean CNC score for each query region. Regions for which the CNC score could not be calculated due to gaps in the data were removed from the analysis, resulting in 15,279 known 3’UTRs and 6522 predicted 3’UTRs.

### Calculating gene enrichment

To investigate molecular functions and biological processes significantly associated with a gene list, we performed GO enrichment analysis in R using the clusterProfiler package (v3.14.3)^71^. All genes expressed in brain were used as the background (*n* = 18,036). The list of brain expressed genes was obtained from the SynGO portal^45^. GO terms attaining an enrichment *q* value (false-discovery rate computed using Benjamini–Hochberg method) <0.05 were considered significant. Similarly, SynGO^45^ was used to identify enriched GO terms (*q* value <0.05) associated with synaptic function, using brain expressed genes as the background. To calculate enrichment of genes associated with rare neurogenetic disorders, OMIM^72^ genes related to neurological disorders were used (1948 genes). The list of genes associated with adult-onset neurodegenerative disorders was extracted from Genomic England PanelApp (254 green labelled genes)^73^. A hypergeometric test was used to calculate the enrichment using brain expressed genes as the ‘gene universe’.

### Statistical analysis

All data processing, statistical analysis, and plotting were conducted in R (version 3.6.2) software. All statistical tests were two-sided. The Wilcoxon effect size (*r*) was calculated using the rstatix package (v0.6.0).

### Public datasets used in this study

ER data: http://rytenlab.com/browser/app/vizER

GTEx median gene expression (v6p): https://www.gtexportal.org/home/datasets

Ensembl GTF: https://www.ensembl.org/index.html

Gencode GTF (comprehensive gene annotation): https://www.gencodegenes.org/human/

UCSC Table Browser: https://genome.ucsc.edu/cgi-bin/hgTables

phastCons scores: https://hgdownload.soe.ucsc.edu/downloads.html

Transposable elements: http://www.repeatmasker.org

DNA structural properties: http://bioinformatics.psb.ugent.be/webtools/ep3/?conversion

Poly(A) site atlas: https://www.polyasite.unibas.ch/atlas

ATtRACT database: https://attract.cnic.es/

MEME motif database: https://meme-suite.org/meme/db/motifs

POSTAR2: http://lulab.life.tsinghua.edu.cn/postar2/index.php

SynGO: https://www.syngoportal.org/

OMIM: https://www.omim.org/

Genomics England PanelApp: https://nhsgms-panelapp.genomicsengland.co.uk/

### Reporting summary

Further information on research design is available in the Nature Research Reporting Summary linked to this article.

## Supplementary information

Supplementary Information

Description of Additional Supplementary Files

Supplementary Data 1

Supplementary Data 2

Supplementary Data 3

Reporting Summary

## Data Availability

All the data used and generated in this study is publicly available. The unannotated 3’UTR predictions across 39 GTEx tissues generated in this study are provided in Supplementary Data 1. F3UTER’s training dataset, ER feature matrix and all 3’ intergenic ER predictions are available to download from the F3UTER web app. RNA-seq data used for non-human species: SRA accession numbers—ERP013119 (mouse liver), SRP197261 (fruit fly midgut) and SRP213938 (zebrafish liver). Paired 3’-seq and RNA-seq data: GEO accession number GSE111310.

## Code availability

The code for F3UTER and the analysis associated with this manuscript is maintained on GitHub: https://github.com/sid-sethi/F3UTER^74^. The source code for running the analyses has been provided as a snakemake pipeline for complete reproducibility.
