# Supplementary material for: Leveraging omic features with F3UTER enables identification of unannotated 3’UTRs for synaptic genes
Source: Nat Commun. 2022 Apr 27;13:2270. doi: 10.1038/s41467-022-30017-z (PMC9046390; doi:10.1038/s41467-022-30017-z)
Supplement: Supplementary file 1 — Supplementary Information [file 41467_2022_30017_MOESM1_ESM.pdf]

SUPPLEMENTARY INFORMATION

Leveraging omic features with F3UTER enables identification of unannotated 3'UTRs for synaptic genes

Siddharth Sethi[1,2], David Zhang[3], Sebastian Guelfi[2,4], Zhongbo Chen[2,3,5], Sonia Garcia-Ruiz[2,3,5], Emmanuel O. Olagbaju[3], Mina Ryten[3,5*], Harpreet Saini[1], Juan A. Botia[2,6]

1. Astex Pharmaceuticals, 436 Cambridge Science Park, Cambridge, United Kingdom.
2. Department of Neurodegenerative Disease, Institute of Neurology, University College London, London, UK.
3. Genetics and Genomic Medicine, Great Ormond Street Institute of Child Health, University College London, London WC1E 6BT, UK.
4. Verge Genomics, South San Francisco, CA 94080, USA.
5. NIHR Great Ormond Street Hospital Biomedical Research Centre, University College London, London, UK.
6. Department of Information and Communications Engineering, University of Murcia, Spain.

∗ Corresponding author: Professor Mina Ryten (mina.ryten@ucl.ac.uk)
These authors contributed equally: Mina Ryten, Harpreet Saini and Juan A. Botia

Supplementary Figures

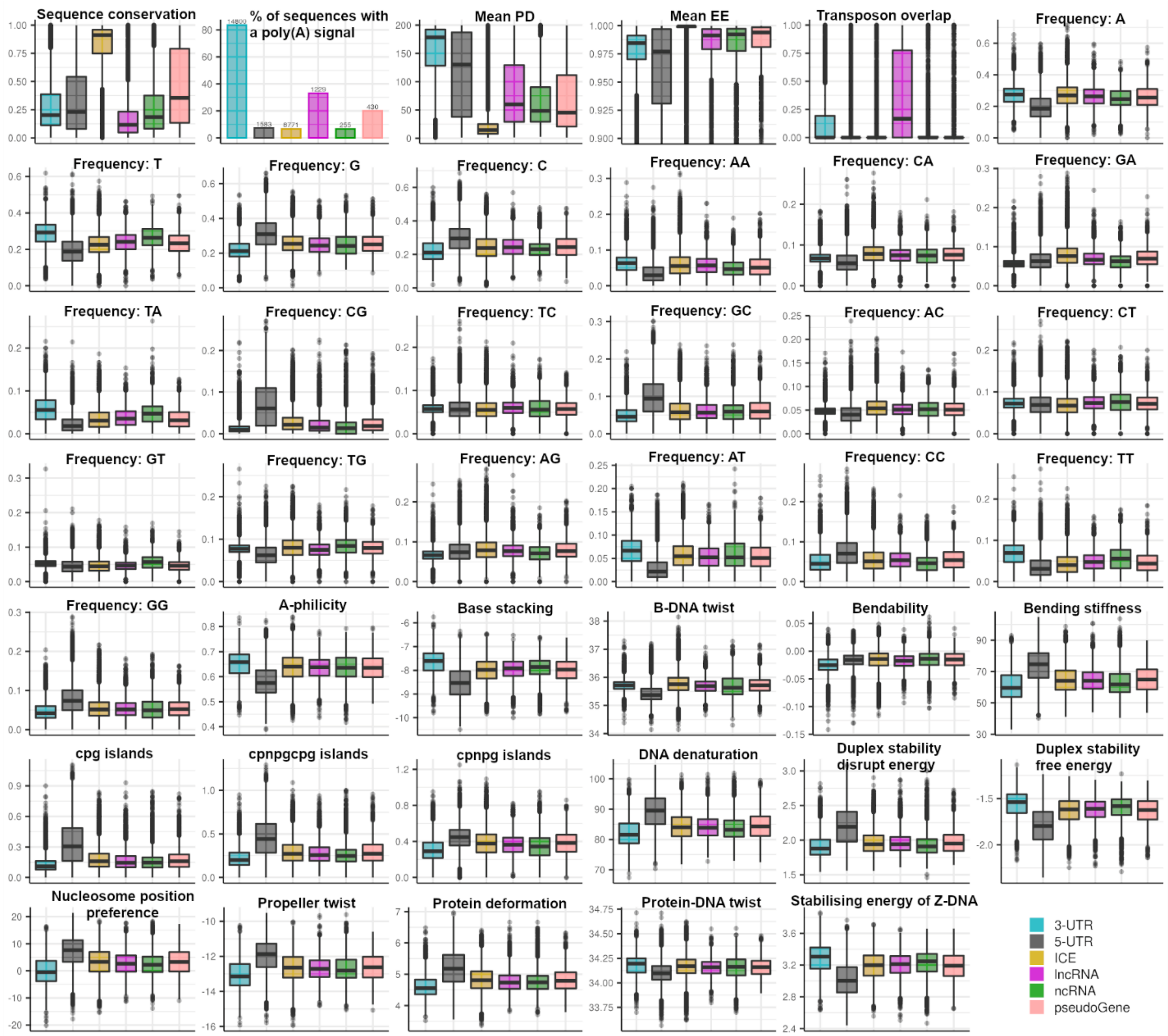

Supplementary Figure 1.

Univariate comparisons of features and genomic classes. Plots show the relationship between quantified features and genomic classes in the training dataset. The genomic classes include: 3'UTRs (n = 17,719), 5'UTRs (n = 21,798), internal coding exons (ICE; n = 130,768), lncRNAs (n = 3,718), ncRNAs (n = 3,819) and pseudogenes (n = 2,146). Box plots show the median value (middle line), 25th and 75th percentile (box), and 1.5 times the interquartile range (whiskers). A Kruskal-Wallis Test was used to compare continuous values of features across the classes, while a two-sided proportion Z-test was used for proportions. For each feature, the comparison across the classes was statistically significant with a $p - value < 2.2 \times 10^{-16}$.

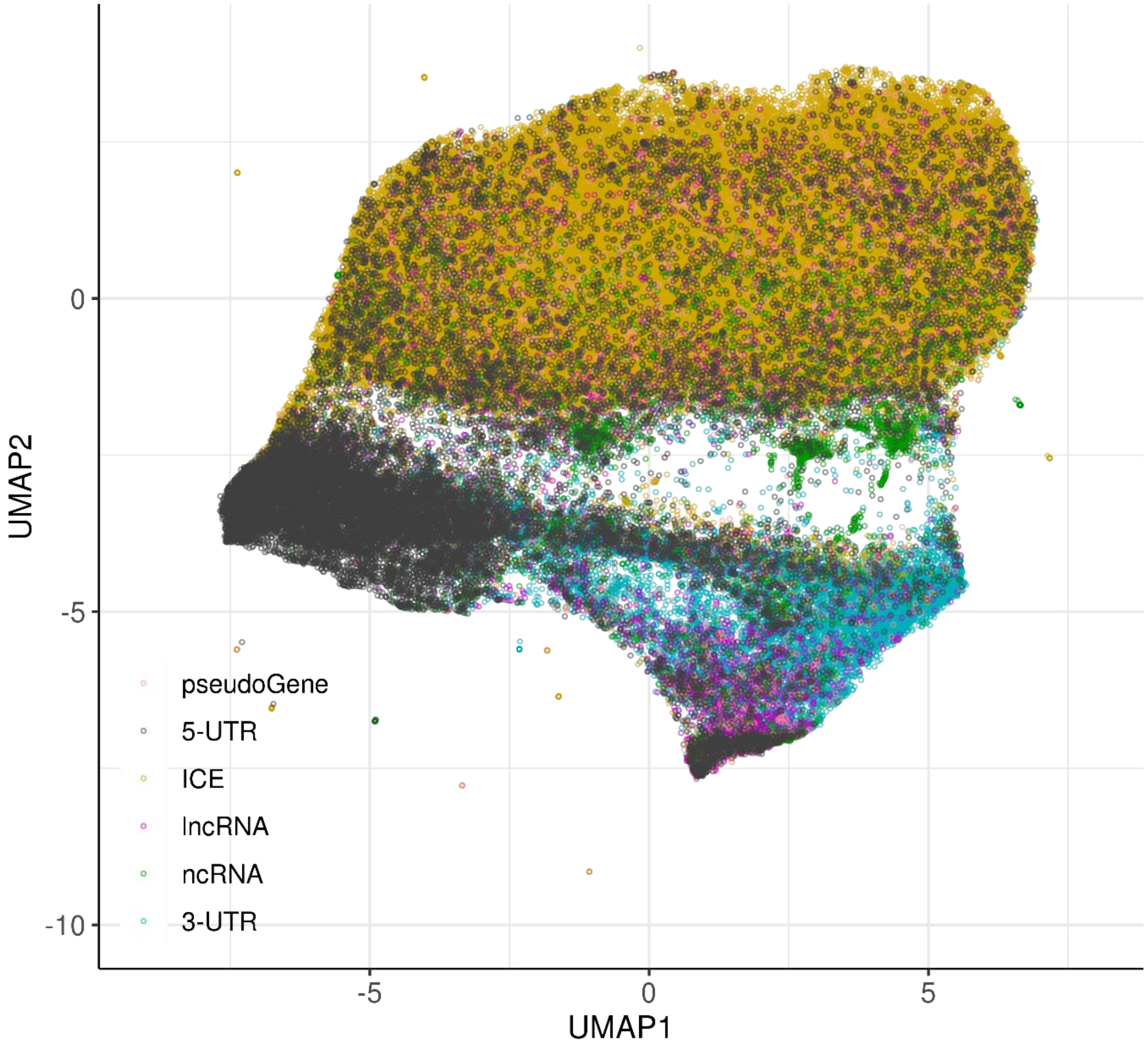

Supplementary Figure 2.

UMAP visualisation of genomic features. UMAP representation of all 41 omic features, with elements labelled by genomic classes.

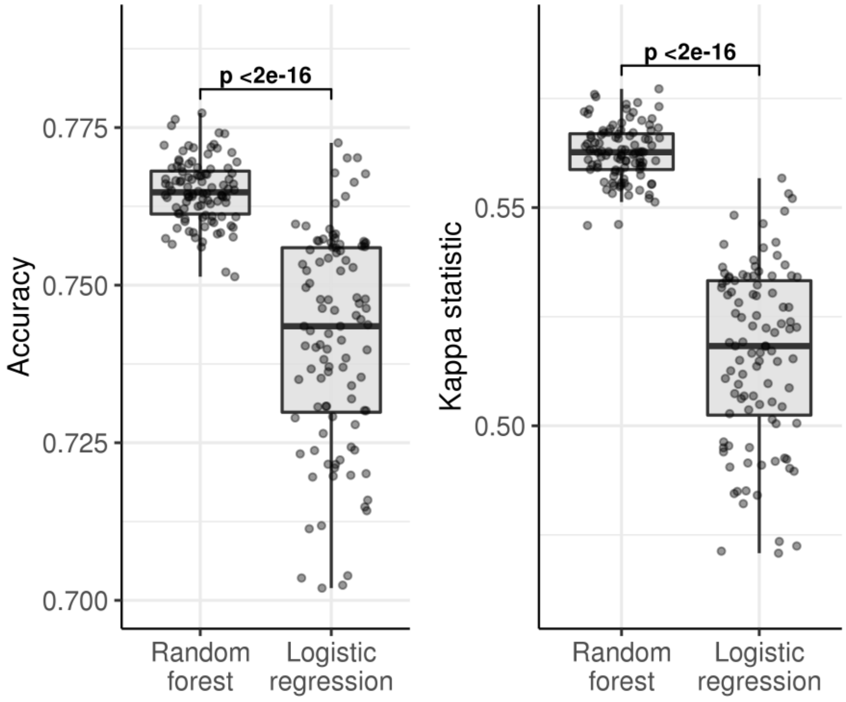

Supplementary Figure 3.

Performance of multinomial classification models measured using 5-fold cross validation repeated 20 times. Box plots comparing the overall accuracy and kappa of random forest multinomial classifier and elastic net multinomial logistic regression model, to classify different genomic classes. Box plots show the median value (middle line), 25th and 75th percentile (box), and 1.5 times the interquartile range (whiskers). p: p-value calculated using two-sided Wilcoxon Rank Sum Test. Accuracy: $p = 4.9 \times 10^{-26}$; Kappa: $p = 7.3 \times 10^{-34}$.

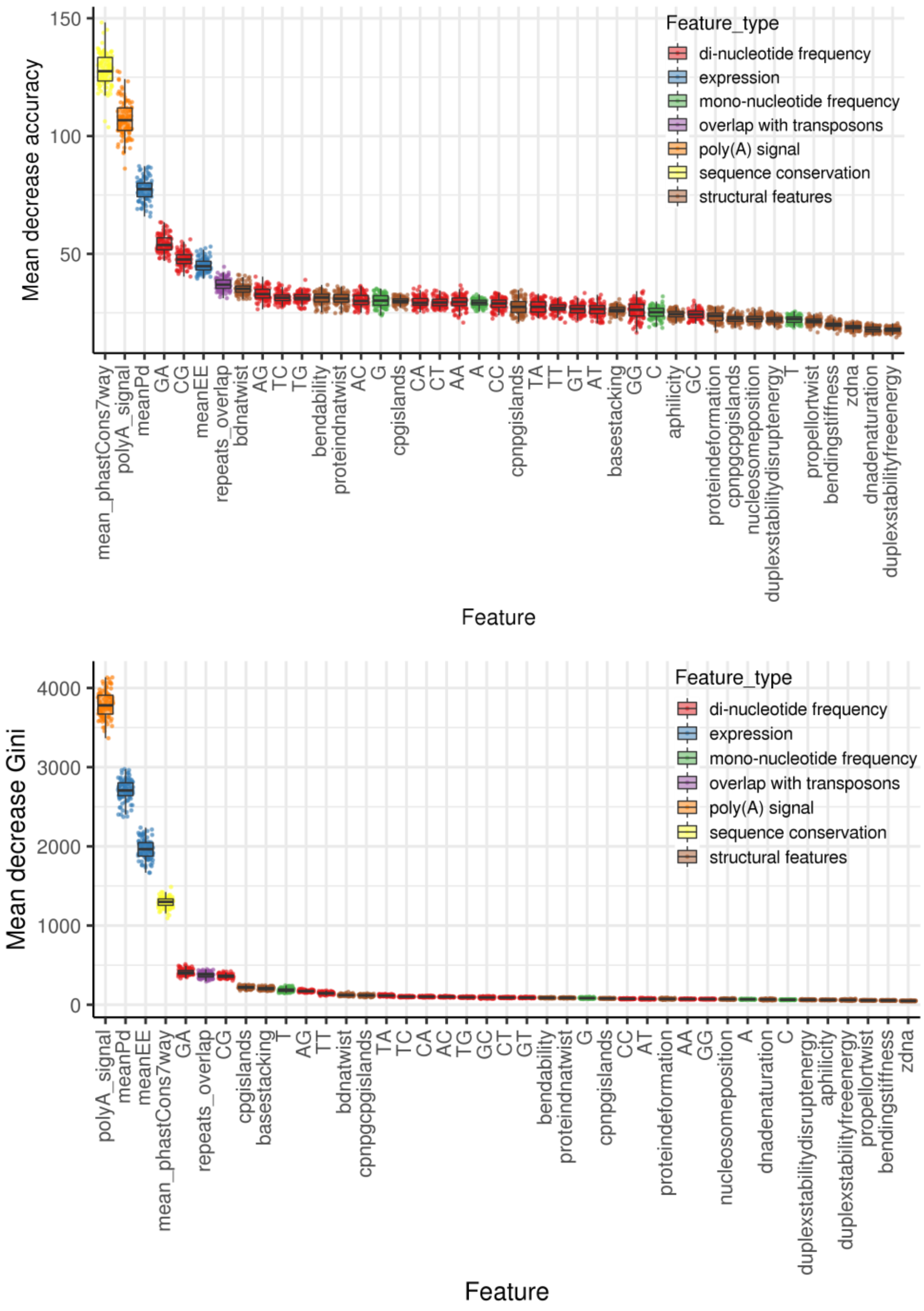

Supplementary Figure 4.

Contribution of features towards 3'UTR classification. Variable importance chart showing the importance of features in classifying 3'UTRs from other transcribed elements in the genome, as measured by mean decrease in accuracy and Gini. The features are ordered in decreasing order of their relative importance and grouped based on their type. The data points for each feature show the values across 5-fold cross validation repeated 20 times (n = 100). Box plots show the median value (middle line), 25th and 75th percentile (box), and 1.5 times the interquartile range (whiskers).

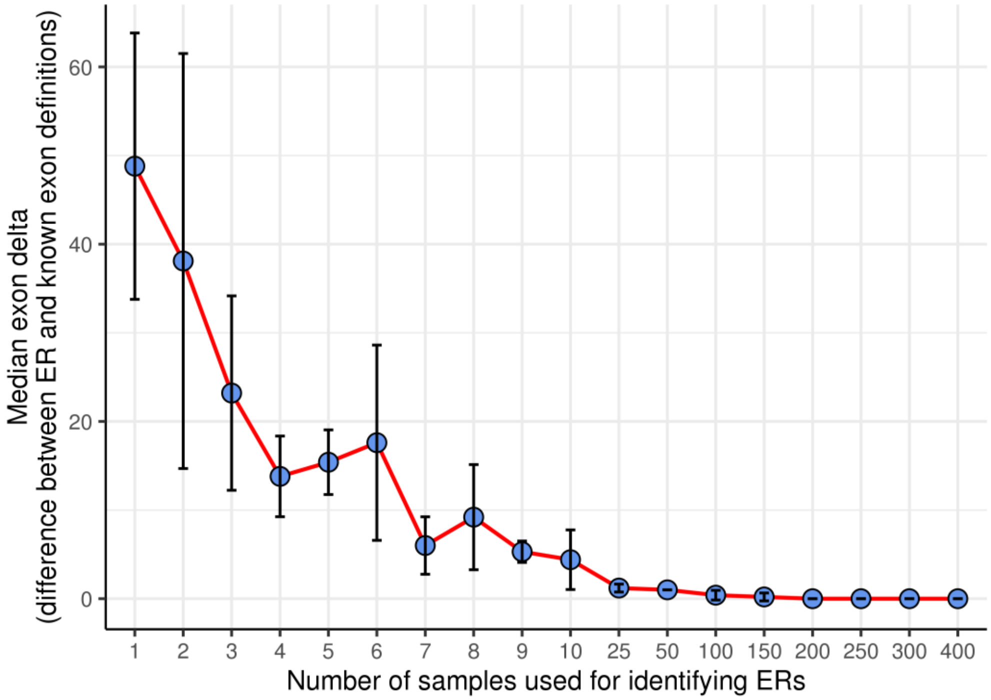

Supplementary Figure 5.

Performance of ER calling on bulk RNA-seq datasets. The line chart shows the accuracy of ER definitions compared to known exons achieved with different number of input RNA-seq samples in whole blood (provided by GTEx). ER calling was performed 5 times for each value of number of samples and for each iteration, the number of samples were randomly selected. The data points show the average “median exon delta” calculated across the iterations and the error bars show the standard deviation.

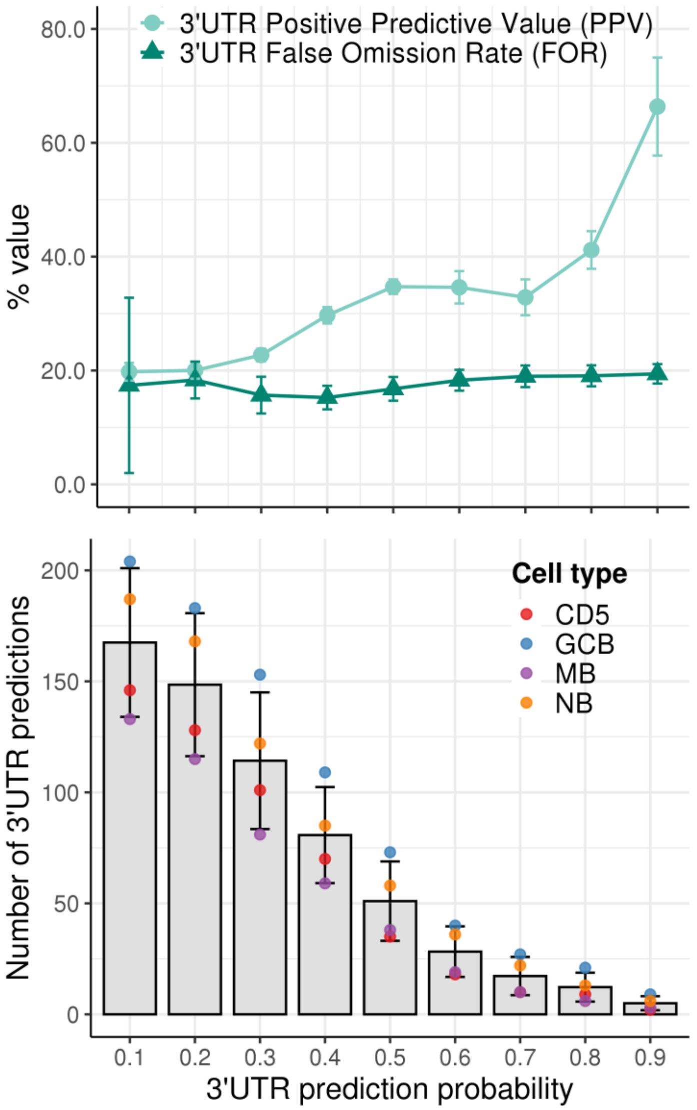

Supplementary Figure 6.

Evaluation of F3UTER 3'UTR predictions at different prediction probability thresholds using 3'-end sequencing data. The line chart at the top shows the PPV and FOR of 3'UTRs predicted by F3UTER at different prediction probability thresholds. The data points represent the average value across the four cell types (CD5, GCB, MB and NB), while the error bars show the standard deviation. The barplot at the bottom shows the number of 3'UTRs predicted by F3UTER at every prediction probability threshold. The bars represent the average number of 3'UTR predictions across the four cell types, while the error bars show the standard deviation. The data points show the exact number of 3'UTR predictions in each cell type.

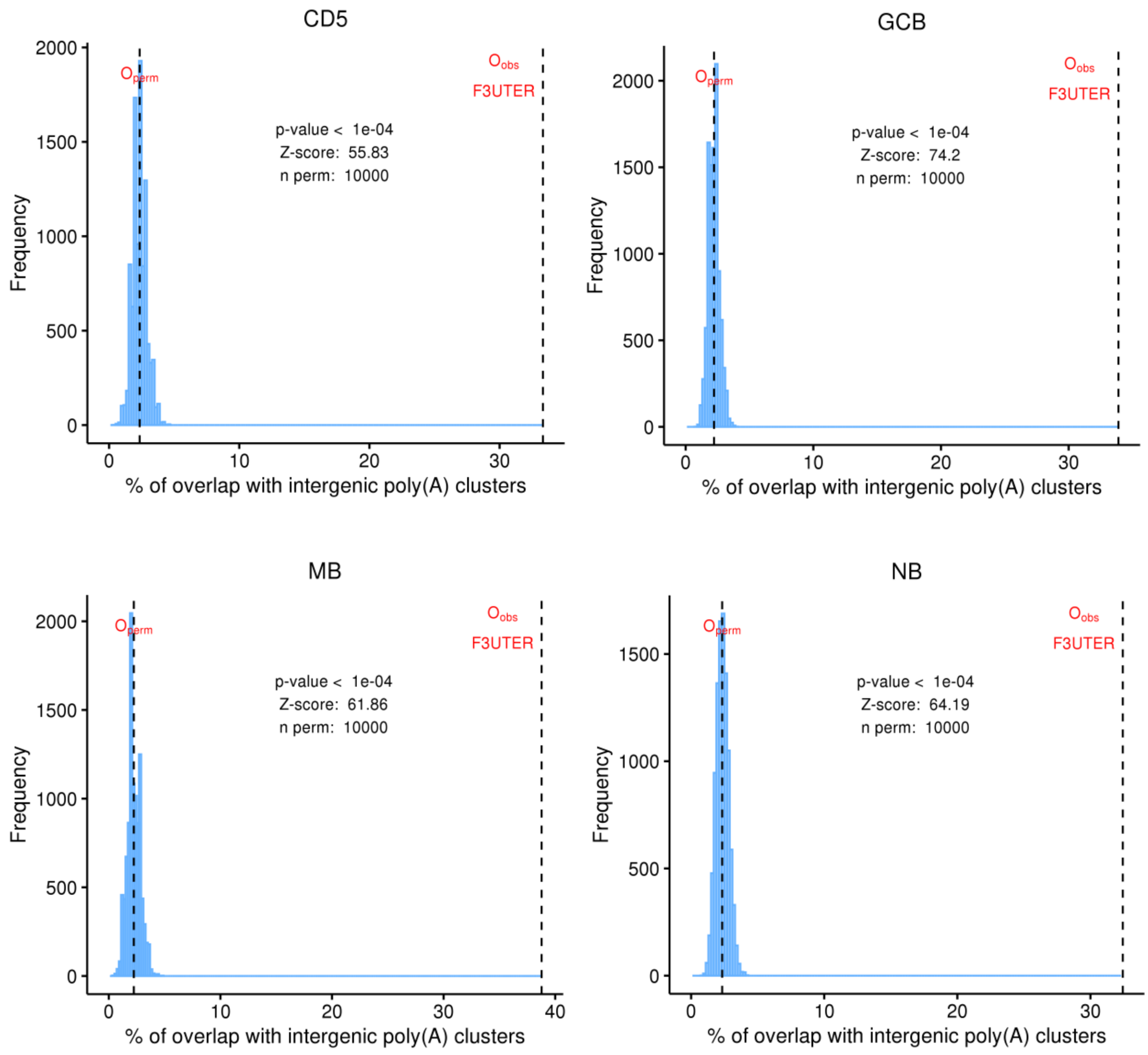

Supplementary Figure 7.

Overlap between randomly selected intergenic ERs and poly(A) sites. Distribution of overlap between randomly selected intergenic ERs and poly(A) sites from 10,000 permutations (n perm). p-value was calculated using a two-sided permutation test. O_{perm}: mean overlap of the permuted distribution; O_{obs}: observed overlap of 3'UTR predictions.

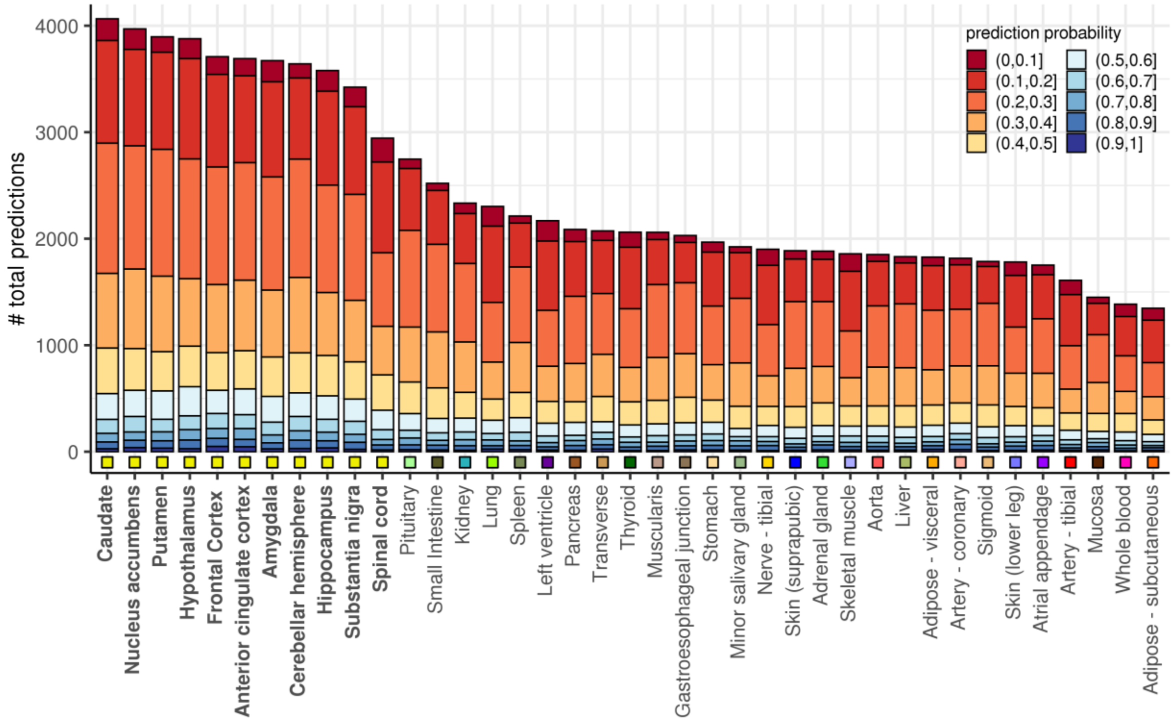

Supplementary Figure 8.

F3UTER predictions across 39 GTEx tissues. Barplot showing the number of predictions in each tissue, grouped and color-coded according to their prediction probability scores. Tissues are sorted in descending order of the total number of predictions in each tissue. The square boxes below the bars are color-coded to group the tissues according to their physiology, and the brain tissues are highlighted in bold.

a

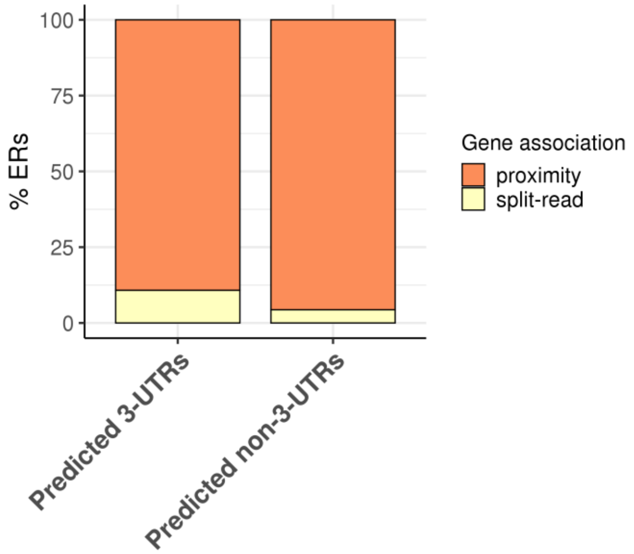

b

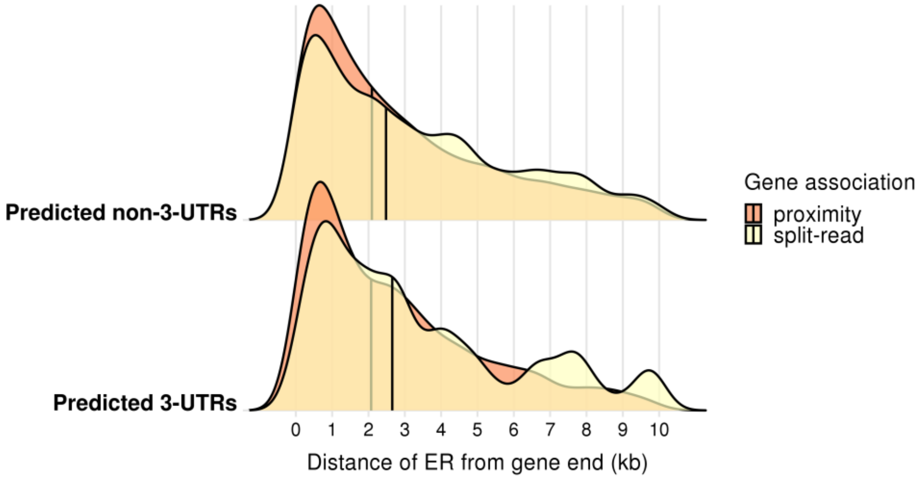

Supplementary Figure 9.

ER-gene associations across 39 GTEx tissues. (a) Barplot showing the proportion of ERs associated to genes based on connecting junction reads or proximity (i.e., nearest expressed gene). The ERs are divided into 3'UTRs and non-3'UTRs based on predictions from F3UTER. Predicted 3'UTRs: n = 7,528; Predicted non-3'UTRs: n = 87,394. **(b)** Distribution of distance between ERs and their associated genes, grouped by the type of gene association and prediction from F3UTER. The vertical solid lines represent the median value of the respective distributions.

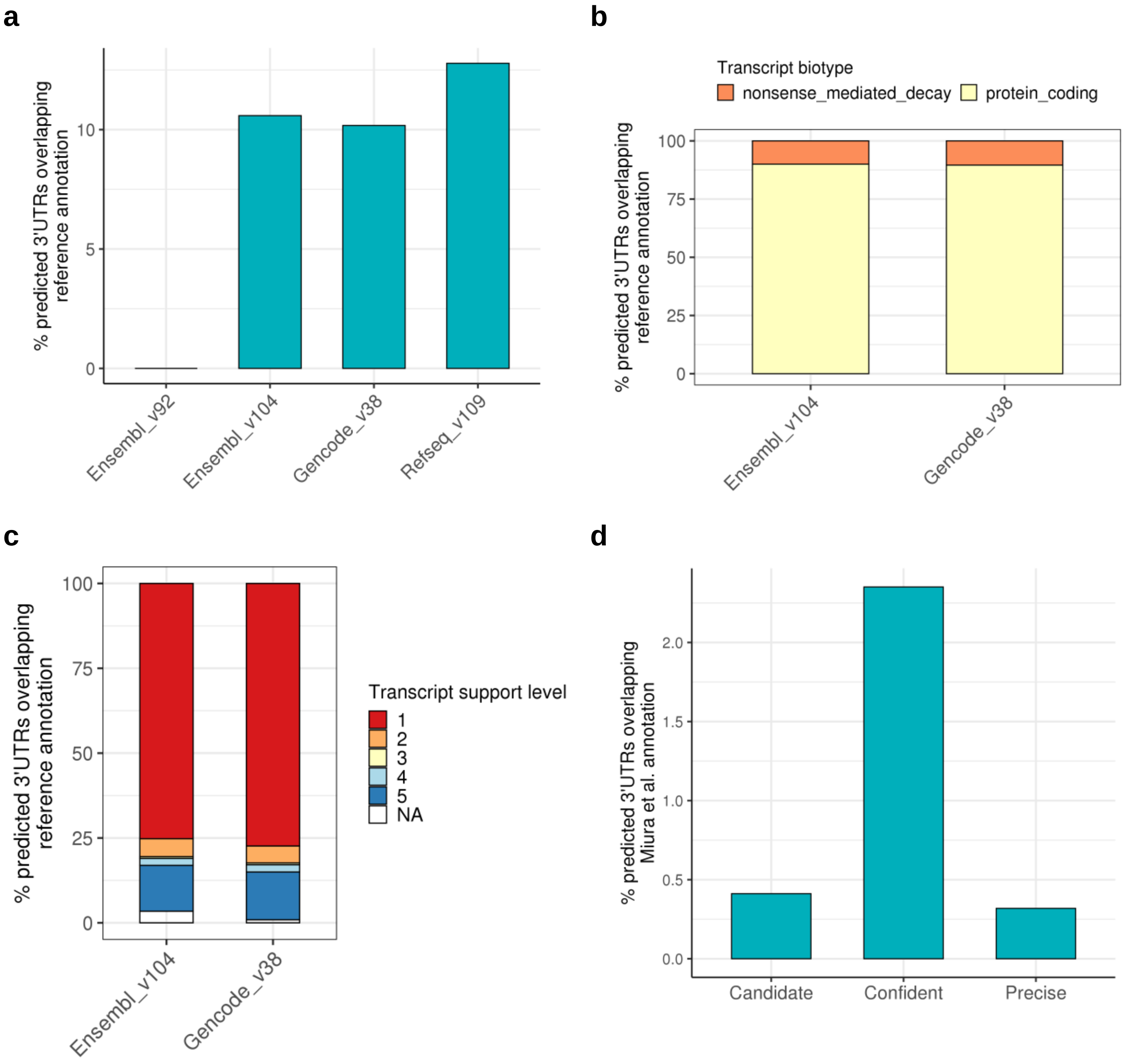

Supplementary Figure 10.

Comparison of predicted unannotated 3’UTRs with reference gene annotation. (a) Bar chart showing the proportion of ERs overlapping annotated 3’UTRs in reference gene annotations. **(b)** Biotype of transcripts associated with annotated 3’UTRs which overlap unannotated ERs. **(c)** Transcript support level of transcripts associated with annotated 3’UTRs which overlap unannotated ERs. **(d)** Bar chart showing the proportion of ERs overlapping predicted 3’UTR extensions (candidate, confident and precise prediction lists) from Miura et al. data.

a

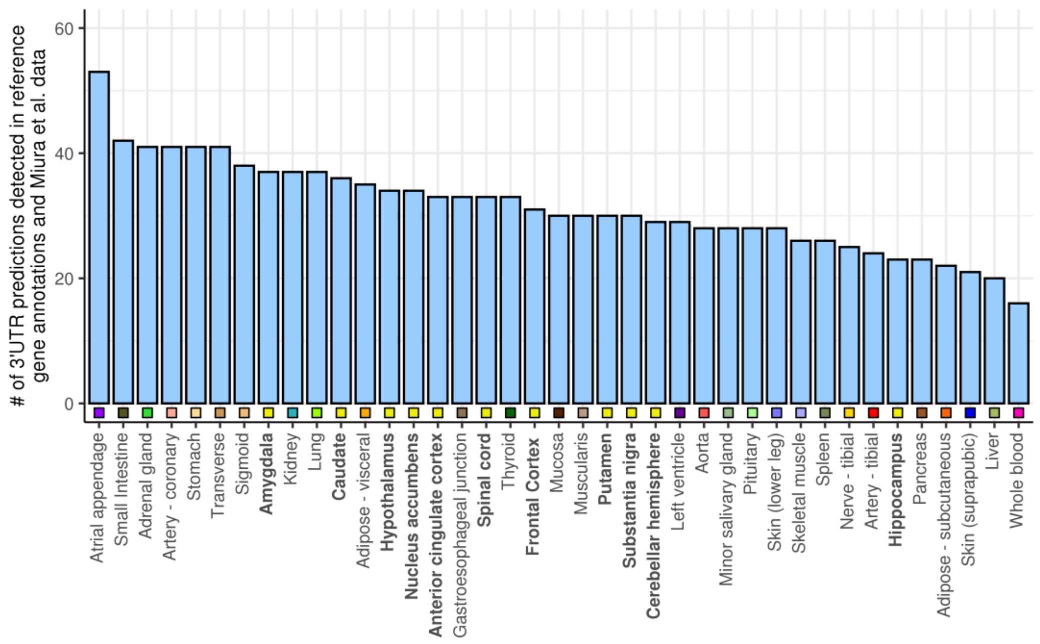

b

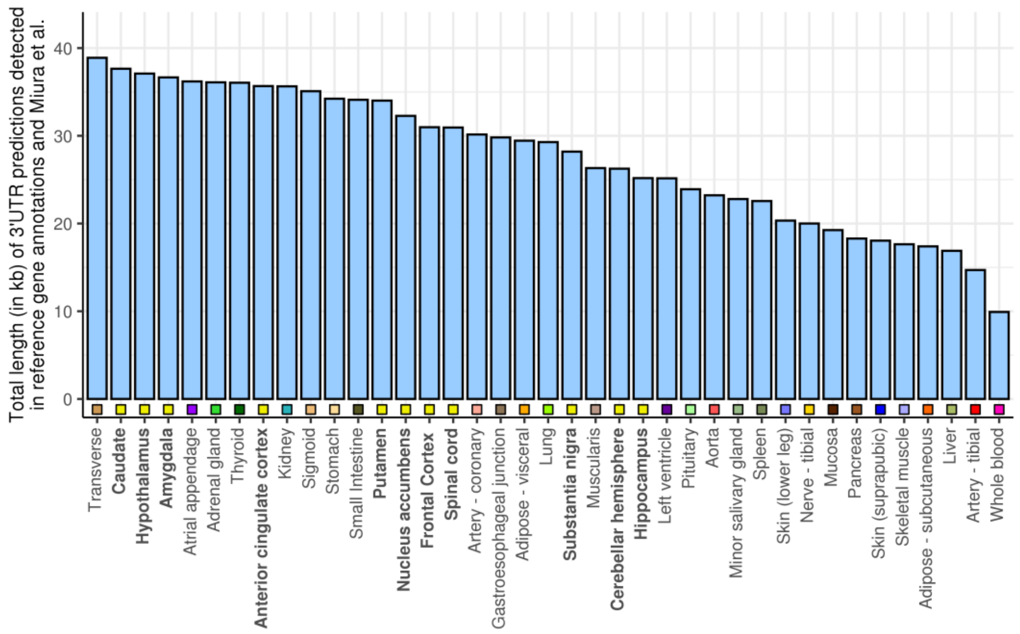

Supplementary Figure 11.

Predicted unannotated 3'UTRs detected in recent reference gene annotations and data from Miura et al.. Barplots showing the **(a)** number and **(b)** genomic space (or total length) of unannotated 3'UTR predictions in each tissue which were detected in either reference gene annotations or in Miura et al. annotations. Tissues are sorted in descending order of the value plotted on the y-axis. The square boxes below the bars are color-coded to group the tissues according to their physiology, and the brain tissues are highlighted in bold.

a

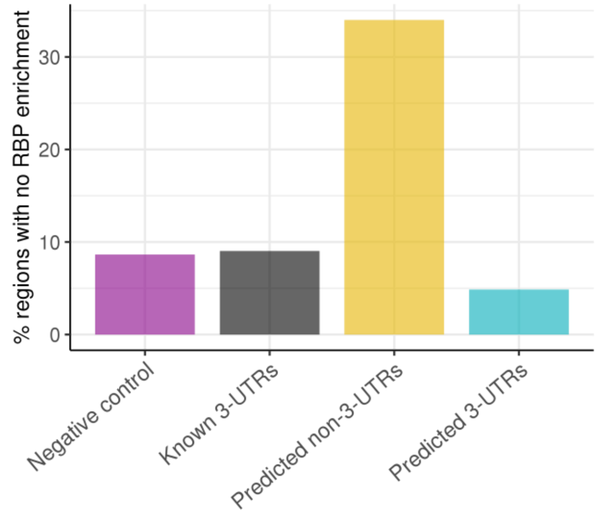

b

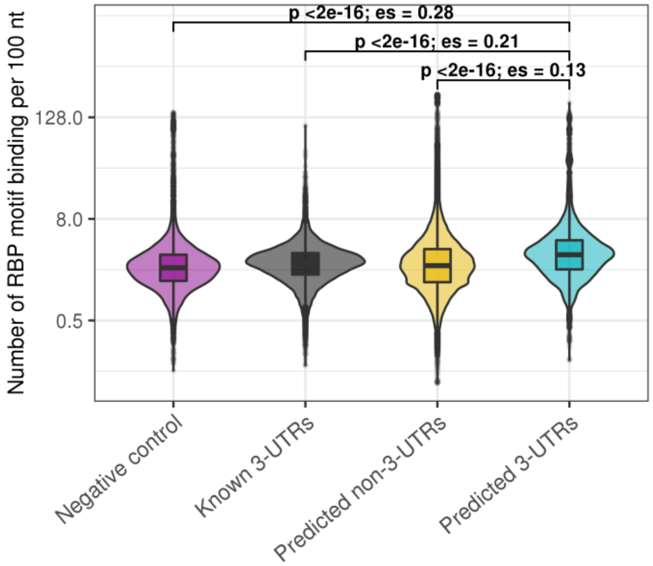

Supplementary Figure 12.

Enrichment of RBP binding motifs in unannotated 3'UTRs. (a) Bar chart showing the percentage of regions in each set where no RBP enrichment was identified. Negative control: n = 651; Known 3'UTRs: n = 1,483; Predicted non-3'UTRs: n = 29,706; Predicted 3'UTRs: n = 366. **(b)** RBP binding enrichment using 97 RBPs from CISBP-RNA database. Only regions with RBP enrichment score greater than zero were displayed. Box plots show the median value (middle line), 25th and 75th percentile (box), and 1.5 times the interquartile range (whiskers). Predicted 3'UTRs vs. non-3'UTRs: $p = 2.8 \times 10^{-282}$; predicted 3'UTRs vs. known 3'UTRs: $p = 9.1 \times 10^{-227}$; predicted 3'UTRs vs. negative control: $p = 1.9 \times 10^{-259}$**.** Negative control: n = 7,392; Known 3'UTRs: n = 15,832; Predicted non-3'UTRs: n = 70,467; Predicted 3'UTRs: n = 7,393; p: p-value calculated using two-sided Wilcoxon Rank Sum Test; es: Wilcoxon effect size (*r*).

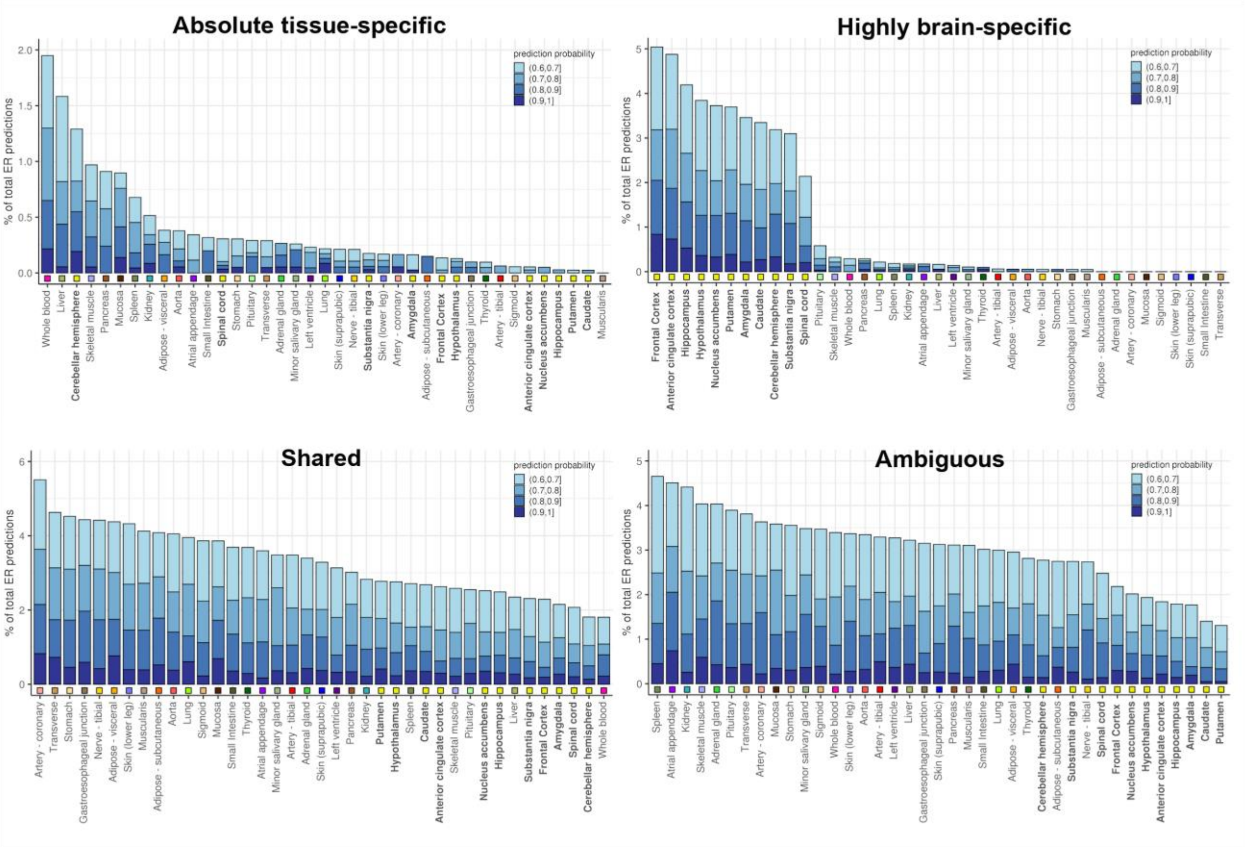

Supplementary Figure 13.

Categorisation of F3UTER predictions based on tissue-specificity. Barplots showing the number of predictions grouped according to their tissue specificity across 39 tissues. Tissues are sorted in descending order of the number of predictions. The square boxes below the bars are color-coded to group the tissues according to their physiology.

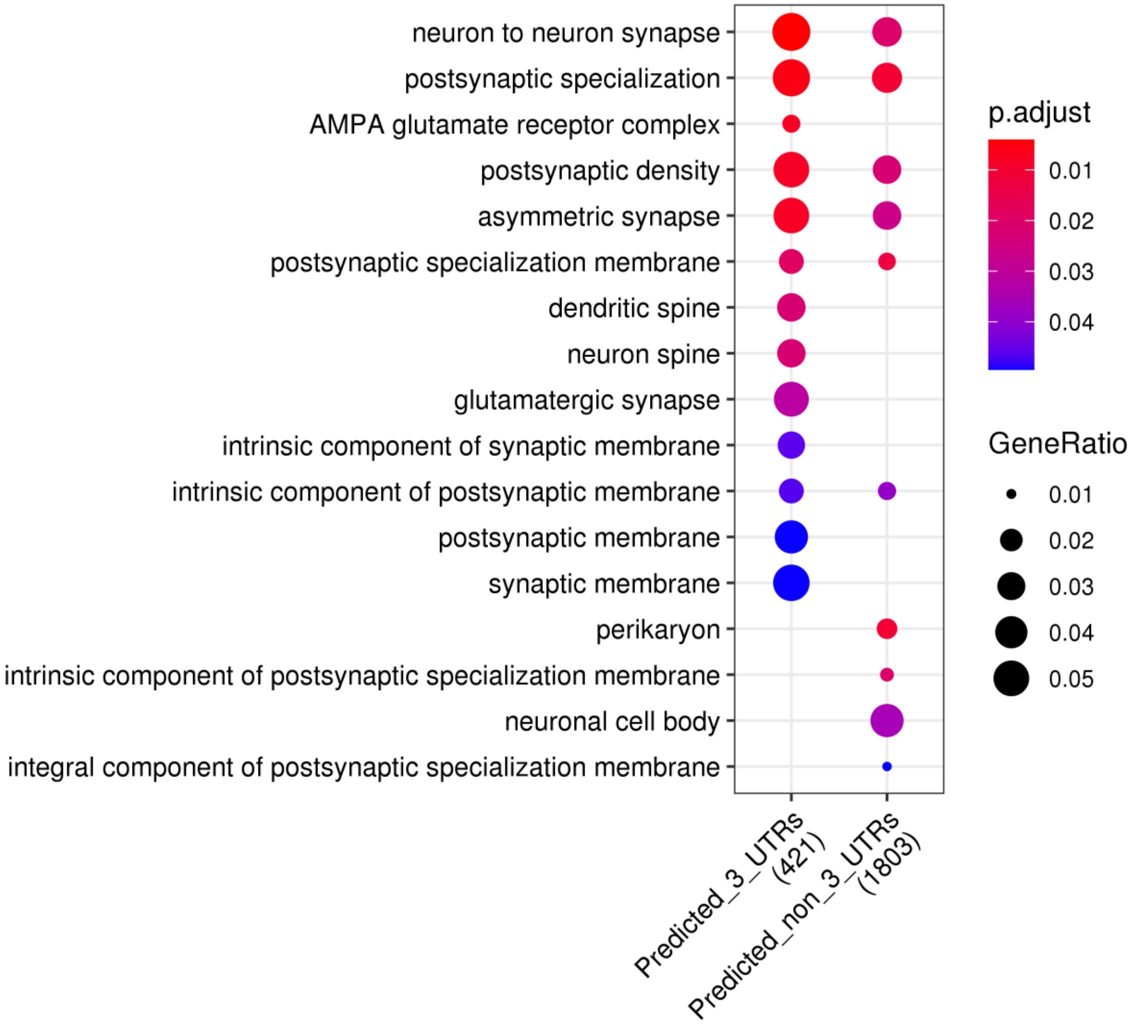

Supplementary Figure 14.

Gene Ontology enrichments of genes associated with highly brain-specific ERs. The plot shows the GO terms enriched amongst the list of genes associated with highly brain-specific 3’UTR predictions and highly brain-specific non-3’UTR predictions.

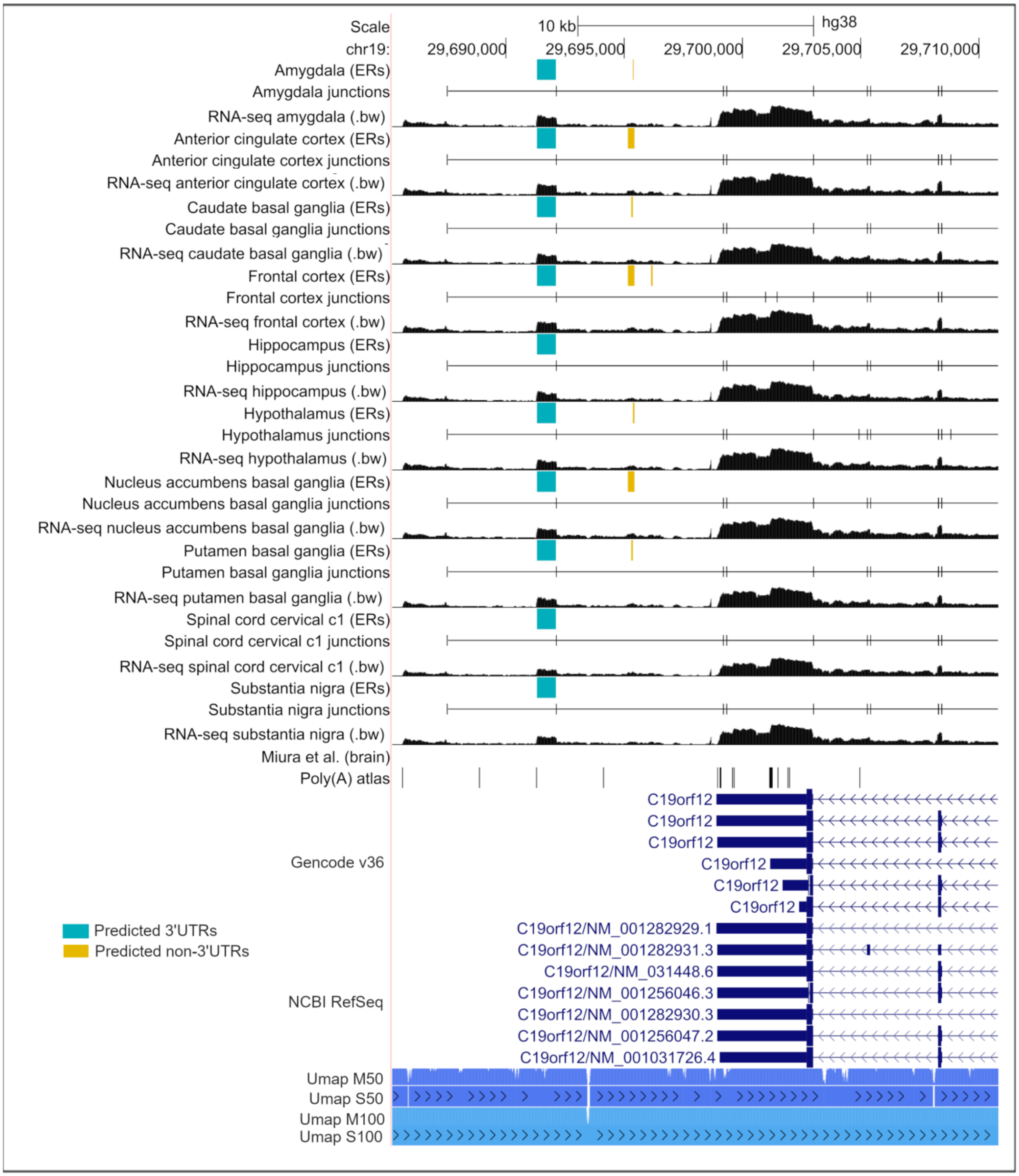

Supplementary Figure 15.

Unannotated 3'UTR associated with *C19orf12* in brain. Genomic view of the *C19orf12* locus displaying intergenic ERs, RNA-seq expression in bigwig (.bw) format, RNA-seq junction reads, 3'UTR extensions from Miura et al., poly(A) sites from the poly(A) atlas, and the human genome

mappability scores from UCSC (Umap). Umap S50 and S100: Single-read mappability for 50- and 100-mers; Umap M50 and M100: Multi-read mappability for 50- and 100-mers.

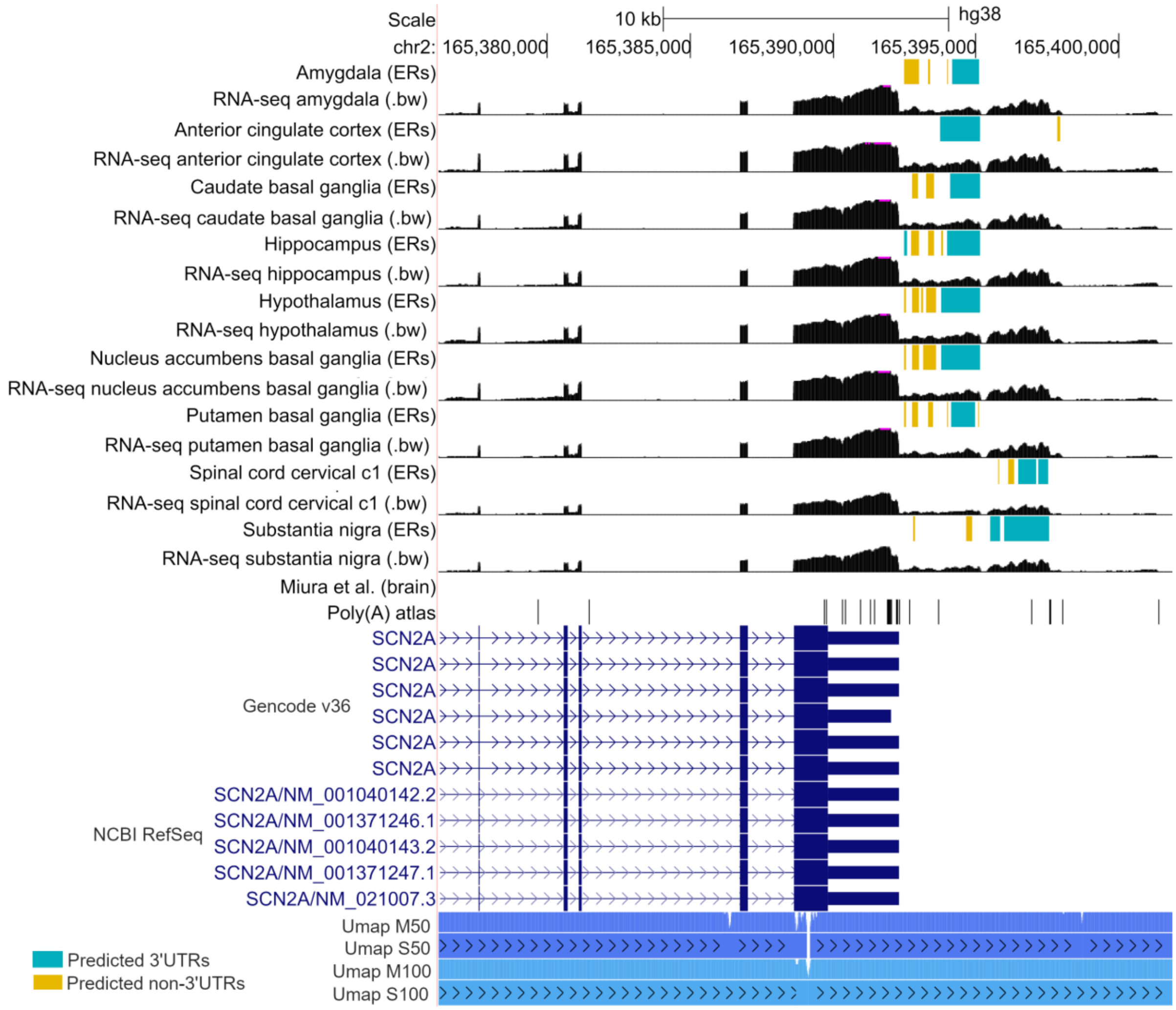

Supplementary Figure 16.

Unannotated 3'UTRs associated with *SCN2A* in brain. Genomic view of the *SCN2A* locus displaying intergenic ERs, RNA-seq expression in bigwig (.bw) format, RNA-seq junction reads, 3'UTR extensions from Miura et al., poly(A) sites from the poly(A) atlas, and the human genome mappability scores from UCSC (Umap). Umap S50 and S100: Single-read mappability for 50- and 100-mers; Umap M50 and M100: Multi-read mappability for 50- and 100-mers.

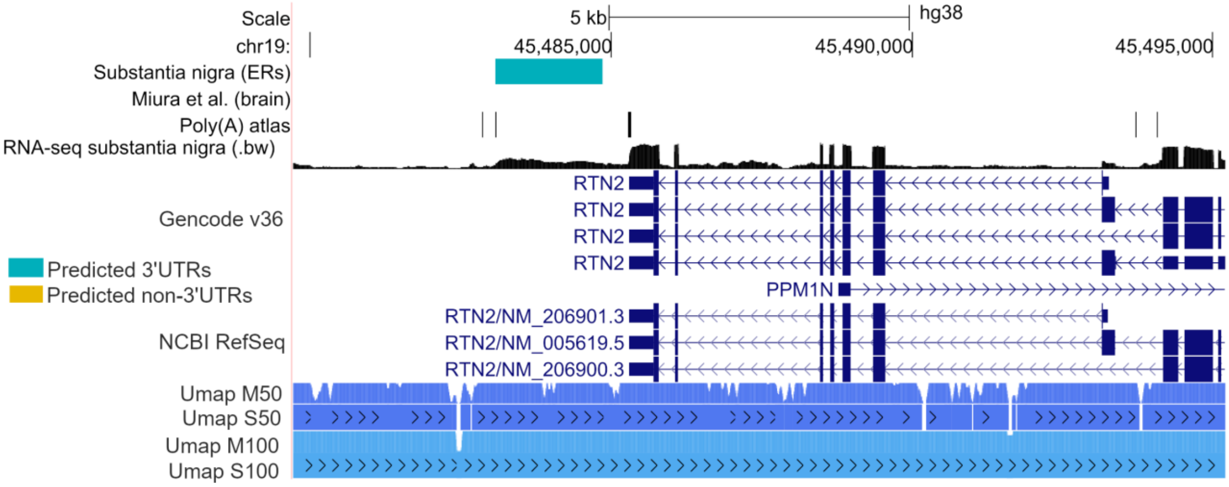

Supplementary Figure 17.

Unannotated 3'UTR associated with *RTN2*. Genomic view of the *RTN2* locus displaying intergenic ERs in substantia nigra, RNA-seq expression in bigwig (.bw) format, RNA-seq junction reads, 3'UTR extensions from Miura et al., poly(A) sites from the poly(A) atlas, and the human genome mappability scores from UCSC (Umap). Umap S50 and S100: Single-read mappability for 50- and 100-mers; Umap M50 and M100: Multi-read mappability for 50- and 100-mers.

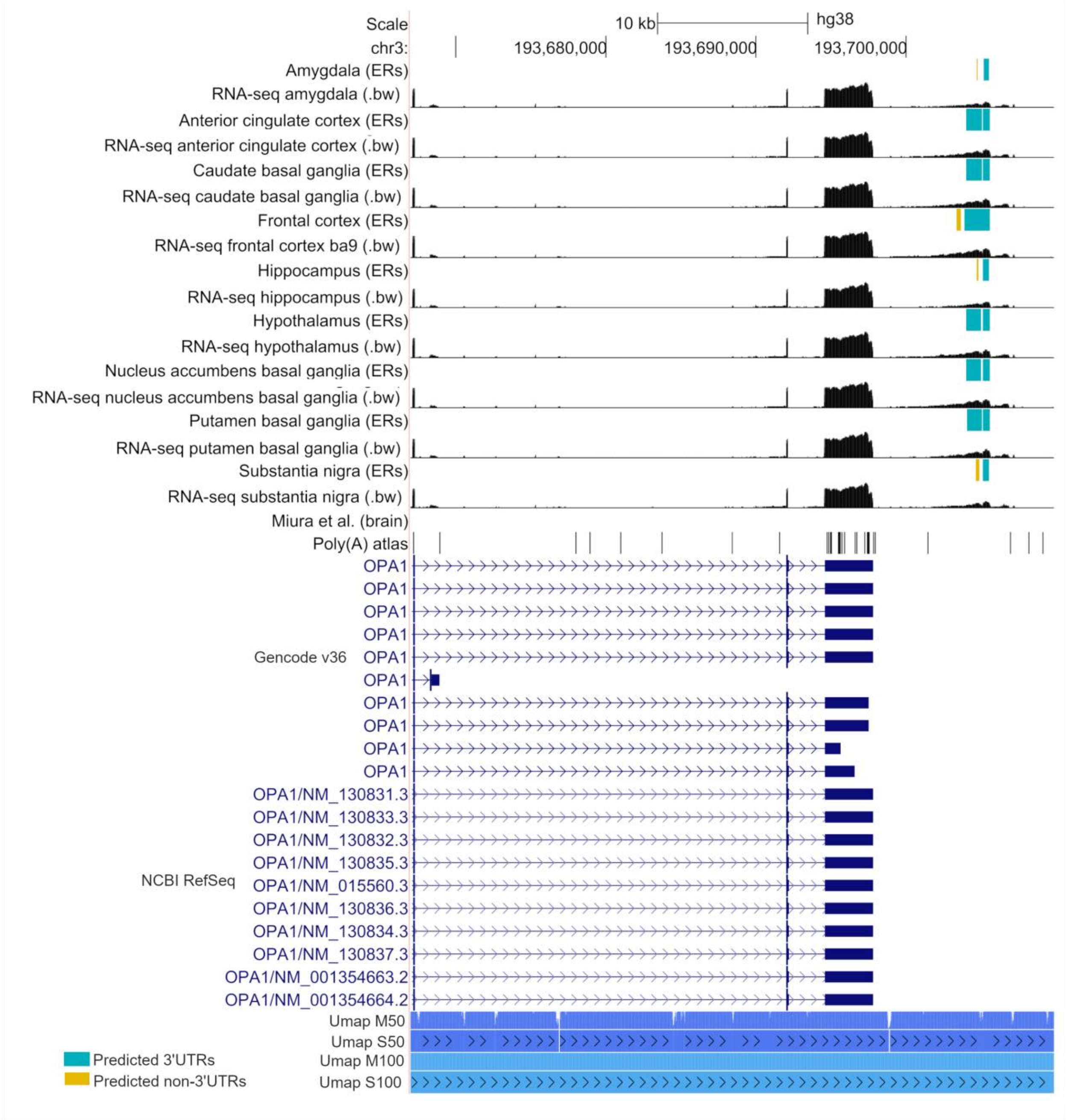

Supplementary Figure 18.

Unannotated 3'UTRs associated with *OPA1* in brain. Genomic view of the *OPA1* locus displaying intergenic ERs, RNA-seq expression in bigwig (.bw) format, RNA-seq junction reads, 3'UTR extensions from Miura et al., poly(A) sites from the poly(A) atlas, and the human genome mappability scores from UCSC (Umap). Umap S50 and S100: Single-read mappability for 50- and 100-mers; Umap M50 and M100: Multi-read mappability for 50- and 100-mers.

Supplementary Table 1

Performance of random forest multinomial classifier to categorise different genomic classes. The performance was evaluated on the validation dataset using 5-fold cross validation repeated 20 times.

**Performance metric** (mean across all cross validation runs)	**3'UTR**	**5'UTR**	**ICE**	**lncRNA**	**ncRNA**	**pseudogene**
Sensitivity	0.777	0.596	0.806	0.556	0.702	0.349
Specificity	0.99	0.973	0.932	0.955	0.962	0.895
Pos Pred Value	0.895	0.754	0.969	0.208	0.289	0.039
Neg Pred Value	0.976	0.946	0.644	0.99	0.993	0.991
Precision	0.895	0.754	0.969	0.208	0.289	0.039
Recall	0.777	0.596	0.806	0.556	0.702	0.349
F1	0.832	0.666	0.88	0.303	0.409	0.07
Prevalence	0.098	0.121	0.727	0.021	0.021	0.012
Detection Rate	0.076	0.072	0.586	0.011	0.015	0.004
Detection Prevalence	0.085	0.096	0.604	0.055	0.052	0.108
Balanced Accuracy	0.883	0.785	0.869	0.756	0.832	0.622

Supplementary Table 2

Performance of elastic net multinomial logistic regression model to categorise different genomic classes. The performance was evaluated on the validation dataset using 5-fold cross validation repeated 20 times.

**Performance metric** (mean across all cross validation runs)	**3'UTR**	**5'UTR**	**ICE**	**lncRNA**	**ncRNA**	**pseudogene**
Sensitivity	0.913	0.386	0.809	0.531	0.146	0.241
Specificity	0.921	0.995	0.931	0.93	0.996	0.907
Pos Pred Value	0.559	0.915	0.969	0.138	0.435	0.031
Neg Pred Value	0.99	0.922	0.648	0.989	0.982	0.99
Precision	0.559	0.915	0.969	0.138	0.435	0.031
Recall	0.913	0.386	0.809	0.531	0.146	0.241
F1	0.693	0.542	0.882	0.219	0.218	0.055
Prevalence	0.098	0.121	0.727	0.021	0.021	0.012
Detection Rate	0.09	0.047	0.588	0.011	0.003	0.003
Detection Prevalence	0.161	0.051	0.607	0.08	0.007	0.095
Balanced Accuracy	0.917	0.69	0.87	0.731	0.571	0.574

Supplementary Table 3

Performance of F3UTER to categorise 3'UTRs evaluated using 5-fold cross validation repeated 20 times.

**Performance metric** (mean across all cross validation runs)	**Training dataset**	**Validation dataset (hold out)**
Sensitivity	0.999018	0.924479
Specificity	0.963189	0.958613
Pos Pred Value	0.747737	0.709312
Neg Pred Value	0.999889	0.99147
Precision	0.747737	0.709312
Recall	0.999018	0.924479
F1	0.855297	0.802707
Prevalence	0.098456	0.098456
Detection Rate	0.09836	0.091021
Detection Prevalence	0.131547	0.128333
Balanced Accuracy	0.981103	0.941546
Accuracy	0.966716	0.955252
Kappa	0.836932	0.777965
AccuracyLower	0.965777	0.953066
AccuracyUpper	0.967637	0.957365
AccuracyNull	0.901544	0.901544
AccuracyPValue	0	3.78E-280
McnemarPValue	0	1.02e-128

Supplementary Table 4

Validation data for evaluating F3UTER's performance in non-human species.

**Validation regions for F3UTER (TPM > 0.1)**	***M.musculus***	***D.rerio***	***D.melanogaster***
5'UTRs	13,538	21,122	13,163
ICEs	83,860	149,593	23,459
Pseudogenes	355	760	202
3'UTRs	11,033	18,835	10,693
**Total**	108,786	190,310	47,517

Supplementary Table 5

RNA-seq datasets used for calculating transcriptomic features associated with validation regions in non-human species.

**Species**	**Tissue**	**Genotype**	**Sample accession number (SRA)**	**# samples**	**Project accession number (SRA)**
Mouse	Liver	Wild type	ERR1121517 - 533	17	ERP013119
Fruit fly	Midgut	Wild type	SRR9032677 - 679	3	SRP197261
Zebrafish	Liver	Wild type	SRR9662023 - 024	2	SRP213938

Supplementary Table 6

Performance of F3UTER to classify 3’UTRs in non-human species

**Performance metric**	**M.musculus**	**D.rerio**	**D.melanogaster**
Sensitivity	0.837	0.688	0.624
Specificity	0.952	0.913	0.87
Pos Pred Value	0.663	0.462	0.6
Neg Pred Value	0.981	0.964	0.881
Precision	0.663	0.462	0.6
Recall	0.837	0.688	0.624
F1	0.74	0.553	0.612
Prevalence	0.101	0.098	0.239
Detection Rate	0.085	0.067	0.149
Detection Prevalence	0.128	0.146	0.248
Balanced Accuracy	0.894	0.8	0.747
Accuracy	0.94	0.891	0.811
Kappa	0.707	0.493	0.487
AccuracyLower	0.939	0.889	0.807
AccuracyUpper	0.942	0.893	0.816
AccuracyNull	0.899	0.902	0.761

Supplementary Table 7

Paired 3'-seq and RNA-seq datasets analysed from Singh et al. (GSE111310)

Sample	Data type	Derived from	Sample name	# samples	Accession number (GEO)
GCB	RNA-seq	Tonsil	GC1, GC2, GC3, GC4	4	GSM3028302, GSM3028303, GSM3028304, GSM3028305
MB	RNA-seq	Tonsil	M2, M5	2	GSM3028315, GSM3028319
CD5+	RNA-seq	Tonsil	CD5B3, CD5B4	2	GSM3028307, GSM3028308
NB	RNA-seq	Tonsil	NB3, NB4, NB5	3	GSM3028312, GSM3028313, GSM3028314
GCB	3'-seq	Tonsil	GC1, GC2	2	GSM3028281, GSM3028282
MB	3'-seq	Tonsil	M1, M2	2	GSM3028279, GSM3028280
CD5+	3'-seq	Tonsil	CD5+B3, CD5+B4	2	GSM3028273, GSM3028274
NB	3'-seq	Tonsil	NB3, NB4	2	GSM3028277, GSM3028278

Supplementary Table 8

Performance of F3UTER and other poly(A) site prediction tools on the paired RNA-seq and 3'-end data in four B cell datasets.

Tool	Performance metric	CD5	GCB	MB	NB
F3UTER	Sensitivity	0.121	0.194	0.143	0.191
	Specificity	0.937	0.917	0.938	0.905
	Pos Pred Value	0.333	0.339	0.388	0.324
	Neg Pred Value	0.804	0.839	0.8	0.825
	Precision	0.333	0.339	0.388	0.324
	Recall	0.121	0.194	0.143	0.191
	F1	0.177	0.247	0.209	0.241
	Prevalence	0.206	0.179	0.215	0.192
	Detection Rate	0.025	0.035	0.031	0.037
	Detection Prevalence	0.075	0.103	0.079	0.113
	Balanced Accuracy	0.529	0.556	0.541	0.548
	Accuracy	0.769	0.788	0.767	0.768
	Kappa	0.076	0.134	0.105	0.115
	AccuracyLower	0.737	0.763	0.732	0.741
	AccuracyUpper	0.799	0.811	0.8	0.794
	AccuracyNull	0.794	0.821	0.785	0.808
APARENT	Sensitivity	0.374	0.373	0.333	0.348
	Specificity	0.776	0.803	0.821	0.774
	Pos Pred Value	0.239	0.241	0.3	0.227
	Neg Pred Value	0.868	0.885	0.842	0.861
	Precision	0.239	0.241	0.3	0.227
	Recall	0.374	0.373	0.333	0.348
	F1	0.292	0.293	0.316	0.275
	Prevalence	0.158	0.143	0.188	0.16
	Detection Rate	0.059	0.054	0.062	0.056
	Detection Prevalence	0.248	0.222	0.208	0.246
	Balanced Accuracy	0.575	0.588	0.577	0.561
	Accuracy	0.712	0.742	0.729	0.706
	Kappa	0.122	0.144	0.148	0.1
	AccuracyLower	0.678	0.716	0.692	0.676
	AccuracyUpper	0.745	0.767	0.764	0.734
	AccuracyNull	0.842	0.857	0.812	0.84
GETUTR	Sensitivity	0.808	0.83	0.812	0.839
	Specificity	0.134	0.151	0.164	0.197
	Pos Pred Value	0.156	0.135	0.125	0.169
	Neg Pred Value	0.779	0.848	0.856	0.862
	Precision	0.156	0.135	0.125	0.169
	Recall	0.808	0.83	0.812	0.839
	F1	0.261	0.232	0.217	0.282
	Prevalence	0.165	0.137	0.128	0.163
	Detection Rate	0.134	0.114	0.104	0.137
	Detection Prevalence	0.857	0.846	0.833	0.809
	Balanced Accuracy	0.471	0.491	0.488	0.518
	Accuracy	0.245	0.244	0.247	0.302
	Kappa	-0.022	-0.006	-0.007	0.014
	AccuracyLower	0.214	0.22	0.213	0.273
	AccuracyUpper	0.278	0.27	0.283	0.331
	AccuracyNull	0.835	0.863	0.872	0.837
TAPAS	Sensitivity	0.539	0.622	0.619	0.676
	Specificity	0.309	0.274	0.326	0.293
	Pos Pred Value	0.098	0.083	0.125	0.105
	Neg Pred Value	0.828	0.872	0.846	0.88
	Precision	0.098	0.083	0.125	0.105
	Recall	0.539	0.622	0.619	0.676
	F1	0.166	0.147	0.208	0.182
	Prevalence	0.123	0.096	0.135	0.11
	Detection Rate	0.066	0.06	0.083	0.074
	Detection Prevalence	0.672	0.716	0.667	0.704
	Balanced Accuracy	0.424	0.448	0.472	0.484
	Accuracy	0.337	0.307	0.365	0.335
	Kappa	-0.052	-0.027	-0.021	-0.009
	AccuracyLower	0.303	0.281	0.328	0.306
	AccuracyUpper	0.373	0.335	0.405	0.365
	AccuracyNull	0.877	0.904	0.865	0.89

Supplementary Table 9

Number of samples in each tissue-specific category with RBP enrichment score greater than zero.

	**Shared**	**Absolute tissue-specific**	**Highly brain-specific**
**Negative control**	2665	266	1440
**Known 3-UTRs**	12487	634	686
**Predicted non-3-UTRs**	19485	3873	13215
**Predicted 3-UTRs**	2785	286	1493

Supplementary Table 10

Number of samples in each tissue-specific category included in the CNC analysis

	**Absolute tissue-specific**	**Highly brain-specific**	**Shared**
**Known 3-UTRs**	656	706	12721
**Predicted 3-UTRs**	279	1473	2349

Supplementary Table 11

List of genes associated with highly brain-specific unannotated 3'UTRs which are known to be associated with rare neurogenetic disorders.

**Ensembl Gene ID**	**Gene Name**
ENSG00000131943	C19orf12
ENSG00000137074	APTX
ENSG00000142192	APP
ENSG00000123560	PLP1
ENSG00000011275	RNF216
ENSG00000085382	HACE1
ENSG00000100225	FBXO7
ENSG00000145888	GLRA1
ENSG00000125744	RTN2
ENSG00000173175	ADCY5
ENSG00000092969	TGFB2
ENSG00000115904	SOS1
ENSG00000119878	CRIPT
ENSG00000136531	SCN2A
ENSG00000144320	LNPK
ENSG00000138363	ATIC
ENSG00000114279	FGF12
ENSG00000198836	OPA1
ENSG00000164904	ALDH7A1
ENSG00000196743	GM2A
ENSG00000149483	TMEM138
ENSG00000144535	DIS3L2
ENSG00000142676	RPL11
ENSG00000162928	PEX13
ENSG00000163536	SERPINI1
ENSG00000138777	PPA2
ENSG00000112234	FBXL4
ENSG00000104723	TUSC3
ENSG00000156110	ADK
ENSG00000140740	UQCRC2
ENSG00000143390	RFX5
ENSG00000156052	GNAQ
ENSG00000134532	SOX5
ENSG00000182400	TRAPPC6B
ENSG00000169306	IL1RAPL1
ENSG00000155100	OTUD6B
ENSG00000091483	FH
ENSG00000253729	PRKDC
ENSG00000107186	MPDZ
ENSG00000133731	IMPA1
ENSG00000169933	FRMPD4
ENSG00000156471	PTDSS1
ENSG00000123700	KCNJ2
ENSG00000106633	GCK
ENSG00000196569	LAMA2
ENSG00000148053	NTRK2
ENSG00000182255	KCNA4
ENSG00000160200	CBS
ENSG00000099246	RAB18
ENSG00000148672	GLUD1
ENSG00000125351	UPF3B
ENSG00000100815	TRIP11

Supplementary Table 12

List of RBPs with significantly enriched binding in the brain-specific unannotated 3'UTRs compared to shared unannotated 3'UTRs ($adjusted\ p < 10^{-5}$). The enrichment p-value of the motifs was calculated using AME (from MEME suite) and was adjusted for multiple tests using a Bonferroni correction.

Rank	RBP Name	RBP Ensembl id	p-value	Adjusted p- value
1	HNRNPA2B1	ENSG00000122566	1.18E-12	1.44E-10
2	CELF1	ENSG00000149187	2.63E-11	4.60E-09
3	RBFOX1	ENSG00000078328	1.14E-10	6.97E-08
4	TARDBP	ENSG00000120948	2.63E-10	1.01E-07
5	ERI1	ENSG00000104626	2.67E-09	2.15E-06
6	RBM5	ENSG00000003756	2.03E-08	1.83E-05
7	NOVA2	ENSG00000104967	8.25E-07	5.77E-05
8	KHSRP	ENSG00000088247	2.18E-07	8.76E-05
