# Supplementary material for: Leveraging omic features with F3UTER enables identification of unannotated 3’UTRs for synaptic genes
Source: Nat Commun. 2022 Apr 27;13:2270. doi: 10.1038/s41467-022-30017-z (PMC9046390; doi:10.1038/s41467-022-30017-z)
Supplement: Supplementary file 2 — Description of Additional Supplementary Files [file 41467_2022_30017_MOESM2_ESM.pdf]

Description of Additional Supplementary Files

File Name: Supplementary Data 1
Description: Unannotated 3'UTR predictions (prediction probability > 0.6) across 39 tissues.

File Name: Supplementary Data 2
Description: GO enrichment of genes associated with highly brain-specific unannotated 3'UTR predictions.

File Name: Supplementary Data 3
Description: GO enrichment of RBPs significantly enriched in highly brain-specific unannotated 3'UTRs
