# Supplementary material for: Leveraging omic features with F3UTER enables identification of unannotated 3’UTRs for synaptic genes
Source: Nat Commun. 2022 Apr 27;13:2270. doi: 10.1038/s41467-022-30017-z (PMC9046390; doi:10.1038/s41467-022-30017-z)
Supplement: Supplementary file 6 — Reporting Summary [file 41467_2022_30017_MOESM6_ESM.pdf]

nature research

Corresponding author(s): Mina Ryten

Last updated by author(s): Feb 14, 2022

Reporting Summary

Nature Research wishes to improve the reproducibility of the work that we publish. This form provides structure for consistency and transparency in reporting. For further information on Nature Research policies, see our Editorial Policies and the Editorial Policy Checklist.

Statistics

For all statistical analyses, confirm that the following items are present in the figure legend, table legend, main text, or Methods section.

n/a	Confirmed	
☐	☒	The exact sample size (*n*) for each experimental group/condition, given as a discrete number and unit of measurement
☐	☒	A statement on whether measurements were taken from distinct samples or whether the same sample was measured repeatedly
☐	☒	The statistical test(s) used AND whether they are one- or two-sided<br>*Only common tests should be described solely by name; describe more complex techniques in the Methods section.*
☒	☐	A description of all covariates tested
☐	☒	A description of any assumptions or corrections, such as tests of normality and adjustment for multiple comparisons
☐	☒	A full description of the statistical parameters including central tendency (e.g. means) or other basic estimates (e.g. regression coefficient) AND variation (e.g. standard deviation) or associated estimates of uncertainty (e.g. confidence intervals)
☐	☒	For null hypothesis testing, the test statistic (e.g. *F*, *t*, *r*) with confidence intervals, effect sizes, degrees of freedom and *P* value noted<br>*Give P values as exact values whenever suitable.*
☒	☐	For Bayesian analysis, information on the choice of priors and Markov chain Monte Carlo settings
☒	☐	For hierarchical and complex designs, identification of the appropriate level for tests and full reporting of outcomes
☐	☒	Estimates of effect sizes (e.g. Cohen's *d*, Pearson's *r*), indicating how they were calculated

Our web collection on statistics for biologists contains articles on many of the points above.

Software and code

Policy information about availability of computer code

Data collection	SRA-Toolkit (v2.10.0) was used to download data from SRA. No software was used to collect any other data.
Data analysis	All the data analysis was performed in R version 3.6.2. The following packages were used for analysis: TFBSTools (version 1.24.0); umap (version 0.2.7.0); Caret(version 6.0-85); glmnet (version 4.0-2); randomForest (version 4.6-14); universalmotif (version 1.4.10); clusterProfiler (version 3.14.3); rstatix (version 0.6.0). All the code used to perform analyses in this study is publicly available at https://github.com/sid-sethi/F3UTER. Other standalone tools include: STAR (v2.5); kallisto (v0.45.0); BEDtools (v2.29.2); MEME (v5.1.1).

For manuscripts utilizing custom algorithms or software that are central to the research but not yet described in published literature, software must be made available to editors and reviewers. We strongly encourage code deposition in a community repository (e.g. GitHub). See the Nature Research guidelines for submitting code & software for further information.

Data

Policy information about availability of data

All manuscripts must include a data availability statement. This statement should provide the following information, where applicable:

- Accession codes, unique identifiers, or web links for publicly available datasets
- A list of figures that have associated raw data
- A description of any restrictions on data availability

All the data used and generated in this study is publicly available. The unannotated 3’UTR predictions across 39 GTEx tissues generated in this study are provided in Supplementary Data 1. F3UTER’s training dataset, ER feature matrix and all 3’ intergenic ER predictions are available to download from the F3UTER web app: https://astx.shinyapps.io/F3UTER/.

Publicly available datasets used are:

ER data: http://rytenlab.com/browser/app/vizER
GTEx median gene expression (v6p): https://www.gtexportal.org/home/datasets
Ensembl GTF: https://www.ensembl.org/index.html
Gencode GTF (comprehensive gene annotation): https://www.gencodegenes.org/human/
UCSC Table Browser: https://genome.ucsc.edu/cgi-bin/hgTables
phastCons scores: https://hgdownload.soe.ucsc.edu/downloads.html
Transposable elements: http://www.repeatmasker.org
DNA structural properties: http://bioinformatics.psb.ugent.be/webtools/ep3/?conversion
RNA-seq data used for non-human species: SRA accession numbers – ERP013119 [https://www.ncbi.nlm.nih.gov/sra/?term=ERP013119] (mouse liver), SRP197261 [https://www.ncbi.nlm.nih.gov/sra/?term=SRP197261] (fruit fly midgut) and SRP213938 [https://www.ncbi.nlm.nih.gov/sra/?term=SRP213938] (zebrafish liver)
Paired 3′-seq and RNA-seq data: GEO accession number GSE111310 [https://www.ncbi.nlm.nih.gov/geo/query/acc.cgi?acc=GSE111310]
Poly(A) site atlas: https://www.polyasite.unibas.ch/atlas
ATtRACT database: https://attract.cnic.es/
MEME motif database: https://meme-suite.org/meme/db/motifs
POSTAR2: http://lulab.life.tsinghua.edu.cn/postar2/index.php
SynGO: https://www.syngoportal.org/
OMIM: https://www.omim.org/
Genomics England PanelApp: https://nhsgms-panelapp.genomicsengland.co.uk/

Field-specific reporting

Please select the one below that is the best fit for your research. If you are not sure, read the appropriate sections before making your selection.

☒ Life sciences ☐ Behavioural & social sciences ☐ Ecological, evolutionary & environmental sciences

For a reference copy of the document with all sections, see nature.com/documents/nr-reporting-summary-flat.pdf

Life sciences study design

All studies must disclose on these points even when the disclosure is negative.

Sample size	Our computational method was tested on previously published data in four immune cells (GSE111310). To avoid batch effects, we focused on validating our method on data from the same study and samples derived from the same source. Therefore, we selected four cell types for which paired RNA-seq and 3′-seq data was available from the same source. Multiple replicates were used wherever available.
Data exclusions	No data were excluded from the analysis.
Replication	To verify reproducibility in general, multiple replicates were used wherever available. For our computational model, replication was assessed by: (1) evaluating the performance on random training-validation splits using 5-fold cross validation and on data from non-human species; (2) validating on an independent dataset consisting of four cell lines; and (3) applying our method on a wide range of human tissues having sufficient similarities and differences.
Randomization	Randomization was ensured by using random training-validation splits when training our machine learning model.
Blinding	Our study involved the development of a computational method which we applied to publicly available data sets. Blinding is not required as there are no treatment and control groups in our study.

Reporting for specific materials, systems and methods

We require information from authors about some types of materials, experimental systems and methods used in many studies. Here, indicate whether each material, system or method listed is relevant to your study. If you are not sure if a list item applies to your research, read the appropriate section before selecting a response.

Materials & experimental systems

n/a	Involved in the study	
☒	☐	Antibodies
☒	☐	Eukaryotic cell lines
☒	☐	Palaeontology and archaeology
☒	☐	Animals and other organisms
☒	☐	Human research participants
☒	☐	Clinical data
☒	☐	Dual use research of concern

Methods

n/a	Involved in the study	
☒	☐	ChIP-seq
☒	☐	Flow cytometry
☒	☐	MRI-based neuroimaging

April 2020
